# Curcumin-1,2,3-Triazole Conjugation for Targeting the Cancer Apoptosis Machinery

**DOI:** 10.3390/molecules25133066

**Published:** 2020-07-05

**Authors:** Francesca Seghetti, Rita Maria Concetta Di Martino, Elena Catanzaro, Alessandra Bisi, Silvia Gobbi, Angela Rampa, Barbara Canonico, Mariele Montanari, Dmitri V. Krysko, Stefano Papa, Carmela Fimognari, Federica Belluti

**Affiliations:** 1 Department of Pharmacy and Biotechnology, Alma Mater Studiorum-University of Bologna, Via Belmeloro 6, 40126 Bologna, Italy; francesca.seghetti2@unibo.it (F.S.); rita.dimartino@iit.it (R.M.C.D.M.); alessandra.bisi@unibo.it (A.B.); silvia.gobbi@unibo.it (S.G.); angela.rampa@unibo.it (A.R.); 2 Department for Life Quality Studies, Alma Mater Studiorum-University of Bologna, Corso d’Augusto 237, 47921 Rimini, Italy; elena.catanzaro2@unibo.it; 3 Department of Biomolecular Sciences, University of Urbino Carlo Bo, Via Ca’ Le Suore, 2, 61029 Urbino, Italy; barbara.canonico@uniurb.it (B.C.); mariele.montanari@uniurb.it (M.M.); stefano.papa@uniurb.it (S.P.); 4 Cell Death Investigation and Therapy Laboratory, Department of Human Structure and Repair, Ghent University, 9000 Ghent, Belgium; dmitri.krysko@ugent.be; 5 Cancer Research Institute Ghent, 9000 Ghent, Belgium; 6 Department of Pathophysiology, Sechenov First Moscow State Medical University (Sechenov University), 119146 Moscow, Russia

**Keywords:** apoptosis, cell cycle, cell death, click chemistry, curcumin, flow cytometry, fluorine, privileged structure, triazole

## Abstract

The burden of neoplastic diseases is widely recognized as a severe cause of mortality. The clinical inadequacy of most anticancer therapeutics urgently prompted intense drug discovery efforts toward the identification of new chemical entities endowed with a potent and safe antitumor profile. In this scenario, targeting cancer cells apoptosis machinery has emerged as a relevant strategy, useful for tackling the emergence of drug resistance. On this basis, a small library of naturally inspired hybrid molecules was obtained by combining, through a click chemistry approach, “privileged” synthons such as curcumin scaffold and 1,2,3-triazole building block. Compound **1**, bearing a *para*-fluoro phenyl moiety, showed low-micromolar potency against T acute lymphoblastic leukemia cell growth. More in-depth biologic studies demonstrated, for this analog, cell death-inducing properties associated with its capability to simultaneously activate both the receptor and the mitochondrial apoptosis cascades. This peculiar behavior offers promises for achieving an expanded anticancer effect, namely intense cytotoxic response coupled with reduced predisposition of chemoresistance insurgence. Altogether, this study allowed the identification of compound **1** as a lead compound worth to be progressed as an anticancer drug candidate.

## 1. Introduction

Cancer is expected to become, in every country of the world in the 21st century, the leading cause of death along with a remarkable obstacle for the life expectancy increasing [1]. It ranks as one of the principal life-threatening maladies. Among conventional cancer treatments, chemotherapy is one of the most significant approaches, but severe unwanted effects, namely drug toxicity and chemoresistance, cause a reduction in both clinical outcome and long-term survival rates. Despite decades of research, effective therapeutic interventions for several neoplastic diseases still remain elusive and the identification of new molecules endowed with a potent and safe antitumor effect is an urgent need.

The induction of apoptosis represents a common mechanism through which several therapeutics influence cancer cell death. Apoptosis is a very complex cellular phenomenon that can be triggered by caspases activation by two diverse, but overlapping pathways, the intrinsic and the extrinsic one [2]. The intrinsic pathway, also known as mitochondrial or stress or apoptosis, initiates in response to different stresses, such as microtubule-targeting and DNA-damaging agents and oncogenes activation. It is characterized by disruption of the mitochondrial membrane, as a consequence of the liberation of apoptogenic factors, with cell membrane electrochemical gradient disturbance. In contrast, the extrinsic death receptor pathway originates upon specific interactions between protein ligands, such as tumor necrosis factor (TNF) and FAS ligand, and their specific death receptor, followed by activation of caspase-8. Both routes converge in the activation of common downstream effector caspases (caspase-3, -6 and -7) leading to apoptosis execution [3].

The aptitude of malignant cells to evade apoptosis is a widely recognized hallmark also involved in the emergence of drug resistance, so that, targeting apoptosis has emerged as a valuable antitumor therapeutic strategy [4]. In particular, the capability of an anticancer therapeutic to activate both the receptor and the mitochondrial apoptosis cascades is, on two grounds, a greatly desired effect. On one hand, the simultaneous activation of both pathways promotes a more intense cytotoxic response, if compared to the activation of just one of them. On the other, very often, mutations in cancer cells make them unable to deliver one or the other pathway [3]. Indeed, apoptosis inducers offer promises for achieving, in both normal and malignant cells, a decreased toxicity coupled with an improved therapeutic outcome [5,6].

The anticancer potential of natural products (NPs) has attracted considerable attention in both industry and academia. Many screening programs proved to be particularly fruitful for the identification of lead molecules and promising scaffolds for drug discovery purposes, as documented by the current number of drugs based on NPs in clinical trials or available on the market [7,8]. In particular, compound collections based on or inspired by NP structures are expected to be enriched in bioactivity, modulating multiple biologic processes. Among NPs, the class of molecules bearing an α,β-unsaturated carbonyl function, a well-known Michael acceptor system, has been extensively studied and exploited due to the distinctive capability of interacting with nucleophile sites, namely cysteine and lysine residues, of different target proteins [9].

**Figure 1:**
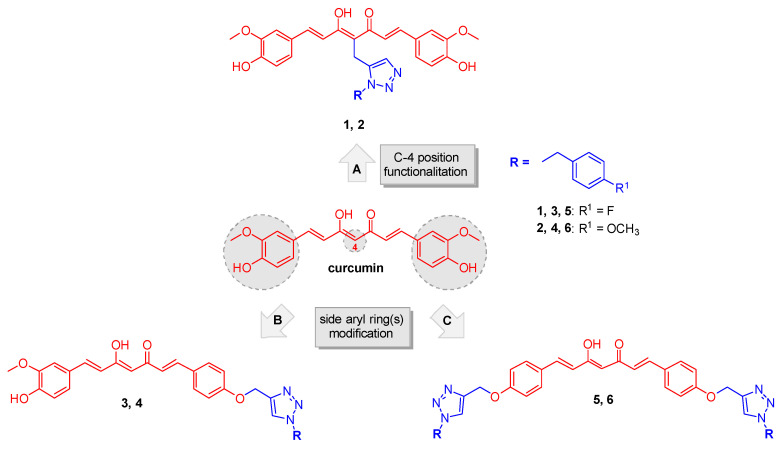
Chemical structure of curcumin and design strategy for obtaining analogs **1**–**6**.

Curcumin (Figure 1), the main bioactive component isolated from the rhizome of Curcuma longa, is one of the most intensely studied phytochemical. Its broad spectrum of beneficial biologic properties including anti-inflammatory, antiproliferative, and antioxidant proved to be particularly suitable for treating complex multifactorial diseases, including cancer [10]. The antitumor effect of curcumin is the consequence of the modulation of a large variety of signaling pathways, likely due to the electrophilic nature of its α,β-unsaturated carbonyl function. For instance, the induction of caspases, principally caspase-3 and -8, endoplasmic reticulum and mitochondrial stress pathways, results in a pro-apoptotic outcome [11].

In spite of the notably favorable antitumor profile, several drawbacks have been reported for curcumin among which poor chemical stability and aqueous solubility that negatively affect its bioavailability and cut the therapeutic window [12].

Curcumin, together with a large number of bioactive NPs, such as epigallocatechin gallate, quinone and rifampicin, has recently been referred to as frequently hitter or pan-assay interfering compound (PAIN) [13,14]. However, a large body of evidence supported that compound reactivity may be strictly related to the structural environment. Thus, derivatives properly designed based on PAIN-based synthons would perform as discriminating bioactive agents rather than reactive chemicals [15,16]. Extensive medicinal chemistry efforts have furnished support to this issue with the identification of a large variety of successful curcumin-related molecules, obtained upon appropriate modifications of its basic framework, also confirming the “privileged” character of this pharmacophore, intrinsically suitable for an efficient interaction with a various biologic targets [17,18,19,20,21]. A fine-tuning of curcumin covalent reactivity, by modulation of the target engagement rate, could represent a strategy through which avoiding the drawback of potential promiscuous reactions [22].

In the light of these concepts the main diarylheptadienone curcumin backbone was suitably modified for achieving specificity while retaining appropriate reactivity. By applying a click chemistry (CC) approach the curcumin scaffold was combined with a 1,2,3-triazole-based synthon bearing a *para*-fluorine and a *para*-methoxy benzyl moieties. In detail, this nitrogen-based heterocycle, obtained through the copper-catalyzed Huisgen 1,3-dipolar cycloaddition, could indeed represent a “privileged” fragment for drug discovery purposes, as it is expected to perform non-covalent interactions with a number of selected targets, offering an opportunity to obtain lead compounds endowed with an enriched efficacy profile. Certainly, this strategy has been largely exploited for the design of lead compounds for therapeutic applications among which the anticancer agents [23,24]. It has been acknowledged that design strategies based on “privileged sub-substructures” combination are a powerful tool to generate promising lead compounds [25]. The fluorine functionalization strategy demonstrated its pivotal role in providing valuable bioactive lead compound in several therapeutic areas [26,27,28].

Based on these considerations, a properly substituted benzyl 1,2,3-triazole structural unit was introduced into different positions of the curcumin scaffold and a small library of analogues was designed and synthesized (routes A, B and C, Figure 1). This strategy encompassed three main structural rearrangements: as a first step, by retaining the whole curcumin structure, functionalization of the central position of the heptatrienone tether was performed (route A, analogs **1** and **2**); then, by maintaining the unmodified central linker, modifications progressively involved the aryl side portions of the parent synthon, i.e., in route B one vanillin-function was kept (analogs **3** and **4**), whereas in route C both the lateral moieties were decorated with the selected triazoles (compounds **5** and **6**). These diversely arranged curcumin analogs **1**–**6** allowed exploring the chemical space around the main skeleton, in the light of drawing the essential features for achieving an improvement of the pharmacological profile, in terms of biologic and physicochemical properties.

The newly synthesized analogs **1**–**6** were first evaluated for their cytotoxic potential, in comparison to curcumin. The best performing candidate identified in this assay was then in-depth investigated to unravel the molecular basis of its anticancer effect in terms of proapoptotic and antiproliferative activities. Particular attention was paid to disqualify a contingent PAIN behavior due to intrinsic autofluorescence.

## 2. Results

### 2.1. Synthesis

**Scheme 1:**
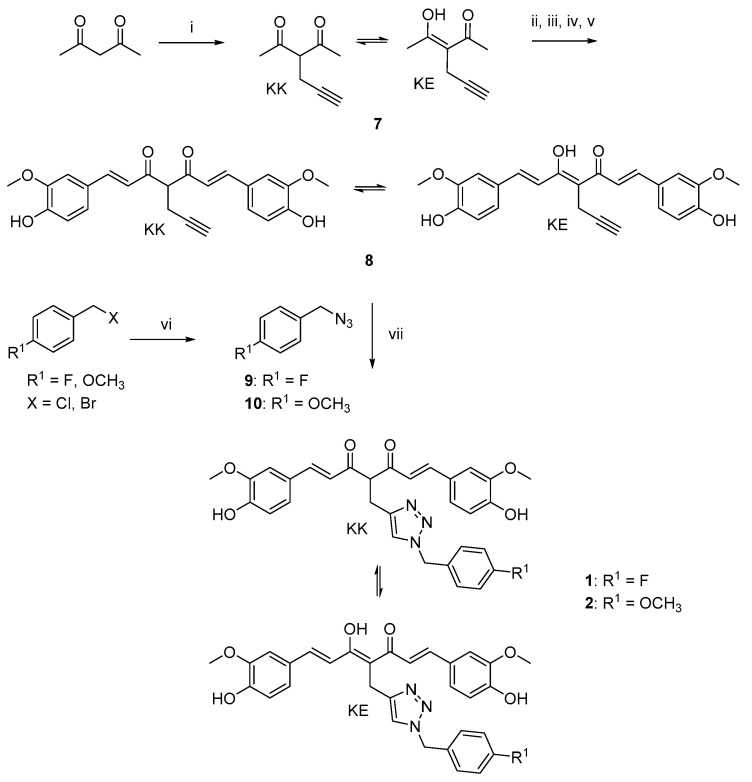
Synthetic strategy for obtaining clicked curcumin-based compounds **1** and **2**. Reagents and conditions: (i) propargyl bromide, K_2_CO_3_, acetone, 80 °C; (ii) B_2_O_3_, DMF; (iii) B(*n*- BuO)_3_; (iv) vanillin, *n*-BuNH_2_, 80 °C; (v) HCl, 80 °C; (vi) NaN_3_, DMF, 60 °C; (vii) TEA, CuSO_4_, (+)-sodium *L*-ascorbate, DMF, r.t.

**Scheme 2:**
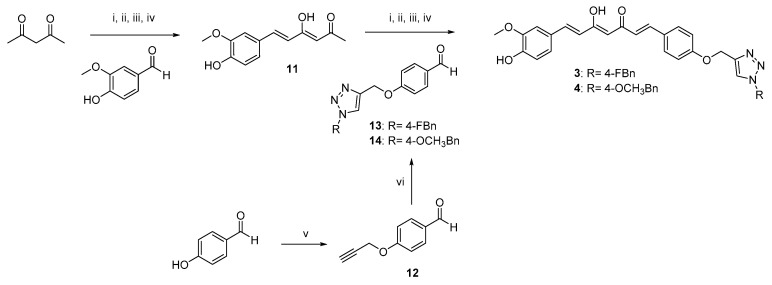
Synthetic strategy for obtaining asymmetric clicked curcumin-based analogs **3** and **4**. Reagents and conditions: (i) B_2_O_3_, DMF; (ii) B(*n*-BuO)_3_; (iii) *n*-BuNH_2_, 80 °C; (iv) HCl, 80 °C; (v) propargyl bromide, K_2_CO_3_, acetone, reflux; (vi**)** selected azide (**9**, **10**), TEA, CuSO_4_, (+)-sodium *L*-ascorbate, DMF, r.t.

**Scheme 3:**
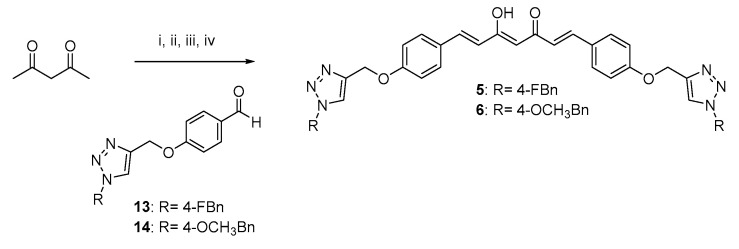
Synthetic strategy for obtaining symmetric clicked curcumin-based analogs **5** and **6**. Reagents and conditions: (i) B_2_O_3_, DMF; (ii) B(*n*- BuO)_3_; (iii) *n*-BuNH_2_, 80 °C; (iv) HCl, 80 °C.

The curcumin-based analogs **1**–**6** were synthetized by exploiting the classical Pabon reaction [29], while, to attain synthons conjugation, the copper-catalyzed azide–alkyne cycloaddition (CuAAC), between the suitable azide (**9** and **10**) and alkyne (**8** and **12**) derivatives was performed (Scheme 1, Scheme 2 and Scheme 3).

As depicted in Scheme 1, pentane-2,4-dione underwent an alkylation reaction with propargyl bromide, in the presence of K_2_CO_3_ as base, to give **7** as mixture of β-keto-enol (KE) and diketo (KK) tautomers; then, a Pabon reaction between **7** and 3-hydroxy-4-methoxy-benzaldehyde (vanillin), in 1:1.8 stoichiometric ratio, allowed to obtain the curcumin-based intermediate **8** as a mixture of KE and KK tautomers. Finally, **8** reacted with the azides **9** and **10** through the Huisgen reaction affording **1** and **2**, respectively as tautomeric couples. Azide intermediates (**9** and **10**) were prepared by a nucleophilic substitution reaction of the appropriate benzyl halides with NaN_3_. For the synthesis of asymmetrical analogs **3** and **4** (Scheme 2) two sequential Pabon reactions were conducted [20], so that pentane-2,4-dione was first reacted with 4-hydroxy-3-methoxybenzaldehyde in a 1:0.9 stoichiometric ratio, to give the semisynthetic intermediate **11** [20] which was then reacted with the functionalized aldehydes **13** and **14**, to attain the desired asymmetrical compounds **3** and **4** in KE tautomeric form. The aldehyde intermediates (**13** and **14**) were prepared via the CuAAC reaction between the appropriate azides (**9** and **10**) and 4-(prop-2-yn-1-yloxy)benzaldehyde (**12**) obtained by reacting 4-hydroxybenzaldehyde and propargyl bromide under Williamson ether synthesis conditions. Finally, Scheme 3 reports the synthetic route for the symmetrical analogs **5** and **6**, starting from pentane-2,4-dione and the selected aldehyde derivatives (**13** and **14**), respectively.

The functionalization of the central curcumin framework promoted the partial conversion of the KE tautomer into the corresponding KK counterpart for derivatives **1**, **2** and **8**, (Scheme 1). In detail, the propynyl-based compound **8**, obtained as a mixture of KE/KK tautomers with a fairly different chromatographic behavior, was carefully isolated by flash column chromatography, thus obtaining **8-KE** in high purity grade. On the other hand, by inserting the benzyl 1,2,3-triazole moiety (**1** and **2**), it was not possible to obtain and isolate each single tautomer as chromatographically different compound. Thus, additional 2D-NMR experiments (^1^H–^1^H COSY, ^1^H–^13^C HSQC, NOESY) were performed on the tautomeric couple of derivative **1** to further confirm compound’s structure (Figures S1–S5, Supplementary Materials). Finally, the introduction of the substituted 1,2,3-triazole ring system on curcumin side aryl ring(s) (**3**–**6**) did not affect the tautomeric conversion, thus maintaining the KE tautomeric form.

### 2.2. Biologic Evaluation

#### 2.2.1. Cytotoxicity

**Figure 2:**
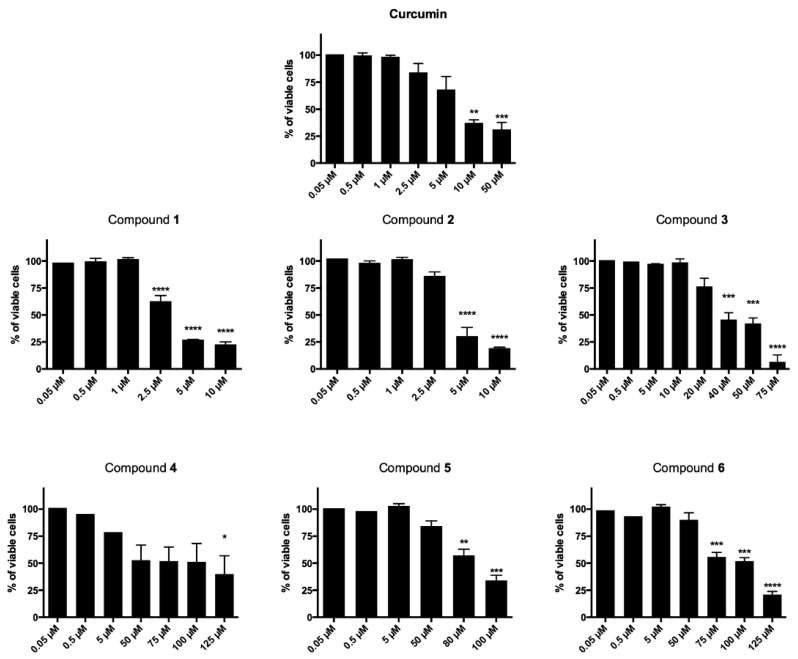
Cytotoxic effects normalized on untreated cells after 48 h of CCRF–CEM treatment with all derivatives and the reference compound curcumin. * *p* < 0.05, ** *p* < 0.01, *** *p* < 0.001, **** *p* < 0.0001 versus untreated cells.

The newly synthesized curcumin-based derivatives **1**–**6** were evaluated for their cytotoxic effect on T acute lymphoblastic leukemia cell line (CCRF–CEM) after 24- and 48-h treatment and data reported are in Table 1 and Figure 2. All derivatives, with the exception of **3**, decreased cell viability in a time-dependent manner (Figure 2). After 24 h treatment, compounds **1**–**3** showed IC_50_ values in the two-digit-µM level, proving to be the most potent of the series, more active than curcumin, for which an IC_50_ higher than 150 µM was observed. Interestingly, after 48-h treatment, a high potency was observed for curcumin and derivatives **1** and **2** (IC_50_ = 10.51, 3.13 and 3.95 µM, respectively). Here, the conjugation of the 1,2,3-triazole-based synthon with the unmodified curcumin framework proved to be particularly favorable for antiproliferative effect that resulted more than three times higher than that of curcumin itself. On the other hand, a mild to low antiproliferative effect was observed for the linearly arranged compounds **3**–**6** (IC_50_ values ranging from 38.00 to 93.40 µM). In detail, when compared to **1** and **2**, molecules **3** and **4**, characterized by a mixed vanillin–triazole fragment combination (i.e., in which one vanillin-based ring was maintained while the other side aryl function was decorated with the selected 1,2,3-triazole-based synthons), showed a substantial decreased activity (IC_50_ = 38.00 and 69.07 µM, respectively) giving the fluorine functionalization the best result. The decoration of both aryl moieties with the 1,2,3-triazoles–synthons turned out into a further reduction of the antiproliferative effect (IC_50_ = 88.94 and 93.40 µM), suggesting that the presence of the vanillin ring is of pivotal importance for inhibition of CCRF–CEM cell proliferation. This results trend was helpful and supportive for the pursued design strategy.

Based on these data, derivative **1**, due to its good low-µM cytotoxic effect was chosen as prototype compound for deeper evaluations aimed at better understanding its mechanism of action. This analog may be interesting from a pharmacokinetic perspective, due to the presence of a fluorine atom that was well-acknowledged to favorably affect pKa, lipophilicity and metabolic stability [30]; moreover, this substituent, could also improve both binding interactions and selective reactivity of the molecule [31], thus providing an enhancement of the therapeutic outcome.

To investigate the anticancer potential of compound **1** on CCRF–CEM cells, a screening pipeline was established in which, the effect on cell-cycle progression was first evaluated. Then, a possible pro-apoptotic effect was determined by a preliminary study on cell membrane structure and integrity. Moreover, mitochondrial potential variation and caspase-8 activity were analyzed to evaluate the impact of **1** on intrinsic and extrinsic apoptosis pathways, respectively. Of note, concentrations that do not exceed its IC_50_ value were employed for these studies, to avoid that non-programmed cell death could take over on a regulated mechanism of action.

#### 2.2.2. Cell-Cycle Progression

**Figure 3:**
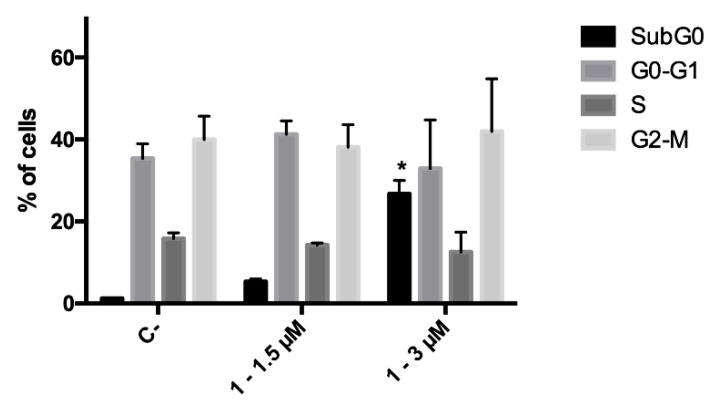
Cell-cycle distribution of CCRF–CEM cells after 24 h treatment with **1**. * *p* < 0.05 versus untreated cells. C-: negative control.

Since during the cytotoxicity assays a decrease in the number of cells was noticed for compound **1**, the ability of this derivative to affect cell proliferation was investigated, and its effect on cell-cycle progression was analyzed. CCRF–CEM cells were treated with 1.5 and 3.0 μM concentrations of **1** for 24 h; at the end of the treatment, no cell-cycle block was recorded at any tested concentration. However, as foretold from cytotoxicity experiments, cell death proved to be the primary mechanism of action of the antitumor potential of analog **1**. Indeed, a significant increase in the fraction of the subG0 phase, which represents the fraction of cells with fragmented DNA, was recorded (Figure 3).

#### 2.2.3. Phosphatidylserine Exposure and Cell Death Analysis

**Figure 4:**
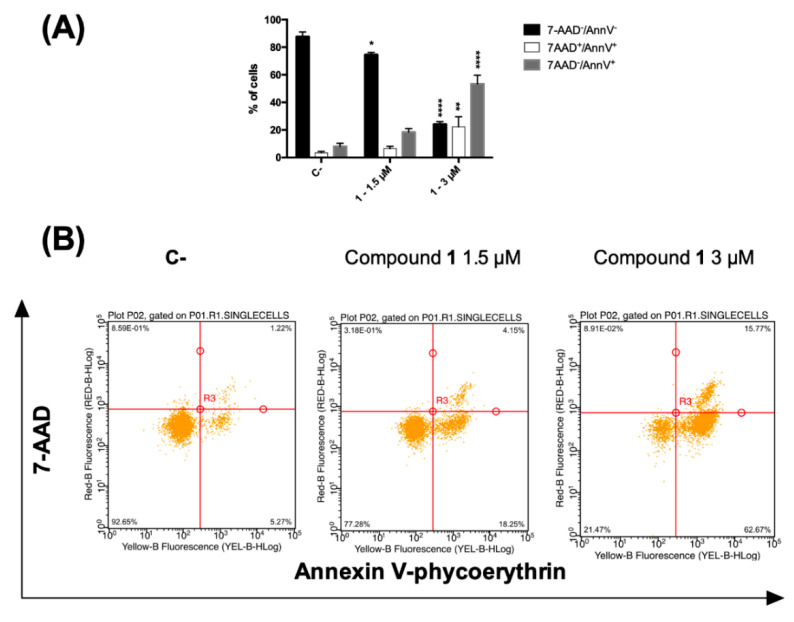
(**A**) Percentage and (**B**) representative dot plot of 7-AAD^−^/annexin V^−^, 7AAD^+^/annexin V^+^ and 7-AAD^−^/annexin V^+^ cells after 24 h treatment of CCRF–CEM cells with compound **1**. * *p* < 0.05, ** *p* < 0.01, **** *p* < 0.0001 versus untreated cells. C-: control.

A loss in plasma membrane asymmetry has been recognized as an early event in dead cells, accompanied by surface exposure of phosphatidylserine (PS) residues, whereas the integrity of the membrane remains unhampered. Surface exposed PS can be detected by its affinity for the phospholipid binding protein annexin V (AV) [32]. On the contrary, both late apoptotic and necroptotic cells suffer the loss of membrane integrity, resulting thus permeable to vital dyes namely the DNA intercalator 7-amino-actinomycin (7-AAD) regarded as marker of membrane integrity [33]. Accordingly, to differentiate cells undergoing early cell death from late cell death (i.e., apoptotic and necroptotic ones), AV/7-AAD binding assay, followed by flow cytometry analysis, was performed allowing to identify cells in the early cell death stage (7AAD^−^/AV^+^), late cell death stage (7AAD^+^/AV^+^) and completely dead cells (7AAD^+^/ AV^−^) [34]. CCRF–CEM cells were treated with 1.5 and 3 µM concentrations of compound **1** for 24 h and stained by using AV and 7-AAD. In general, an increase in the fraction of AV-positive cells was observed. At the highest tested dose, the percentage of cells at the early stages of cell death (AV^+^/7-AAD^−^) was 53.4. However, 22.3% were the cells in the late stages of cell death together with necrotic cells (AV^+^/7-AAD^+^), compared to 8.0% and 3.3%, respectively, recorded for untreated cells (Figure 4A).

The cytotoxic and proapoptotic potential of compound **1** was also tested and confirmed on a different tumor cell line, the acute T cell leukemia Jurkat (Figure S6, Supplementary Materials). Since PS exposure is an event that characterizes both caspase-dependent and -independent types of cell death [35], to exclude that beside apoptosis different forms of regulated cell death concur to compound **1** antitumor potential, we used specific cell death inhibitors: (zVAD-fmk, a pan-caspase blocker), necroptosis [necrostatin-1 s (Nec1s), a RIPK1 inhibitor] or ferroptosis [ferrostatin-1 (fer-1), an inhibitor of reactive oxygen species and lipid peroxidation and deferoxamine (DFO), an iron chelator to characterize apoptosis, necroptosis or ferroptosis, respectively [36,37]. Only zVAD-fmk was able to rescue cells after exposure to compound **1**. Thus, this outcome indicates that compound **1** triggers mere apoptotic cell death.

#### 2.2.4. Mitochondrial Transmembrane Potential

**Figure 5:**
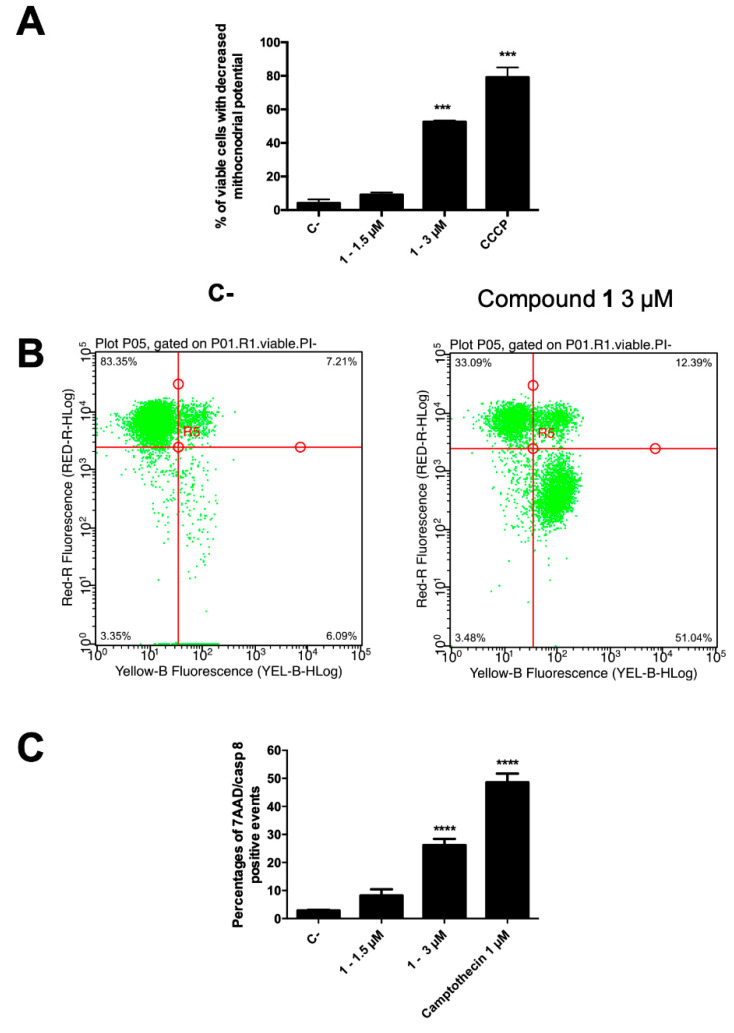
(**A**) Percentage of 7-AAD^+^ and 7-AAD^−^ cells and (**B**) representative dot plot of 7-AAD^−^ cells with decreased mitochondrial potential of **1**-treated CCRF–CEM cells stained with DiIC1(5) and annexin V–phycoerythrin and 7-AAD; (**C**) percentage of apoptotic cells with active caspase-8. *** *p* < 0.001; **** *p* < 0.0001 versus untreated cells. CCCP: carbonyl cyanide 3-chlorophenylhydrazone. C-: control.

In normal cells, the mitochondrial transmembrane potential (MTP, ΔΨm) plays a key role in preserving the function of the respiratory chain, aimed at ATP generation. During apoptosis, MTP disruption occurs, leading to disturbance of the mitochondrial outer membrane permeabilization and collapse of the electrochemical gradient across the membrane [38]. Thus, to evaluate the involvement of the intrinsic pathway, the degree of MTP reduction on CCRF–CEM was determined. The cells, after treatment with compound **1** at 3 µM, were stained by using the membrane permeable lipophilic cation 1,1′,3,3,3′,3′-hexamethylindodicarbo-cyanine iodide DiIC1(5) and its mitochondria level was determined by a flow cytometry analysis. In apoptotic cells, a reduced fluorescence intensity is the indication of MTP disruption, due to the localization of the dye into the cytoplasm. Figure 5A shows the percentage of viable cells suffering a loss of MTP after 1.5 and 3 µM treatment (24 h) with compound **1**. In particular, at 3 µM, 55.9% of **1**-treated cells showed a loss in MTP, clearly demonstrating the involvement of mitochondria pathway-mediated cell death in its anticancer effect. In accordance, around 95% of them were in the early cell death phase, as indicated by AV^+^/7-AAD^−^ assay outcome (Figure 5B).

#### 2.2.5. Caspase-8

Our data on kinetics of PS exposure and MTP suggest that compound **1** induces apoptotic cell death in leukemia cells. In order to identify the type of cell death, caspase-8 activity was measured [34]. The initiator caspase-8 could be considered a connection point between receptor and mitochondrial pathways and its activity allows to discriminate apoptotic cell death from necroptosis [39]. Death receptors stimulation results in caspase-8 activation, which, in turn, by directly cleaving downstream effector caspases, can spread apoptosis signal. On the other side, activation of caspase-8 may result in cleavage of Bid, which translocates to mitochondria to release cytochrome *c*, thereby initiating a mitochondrial amplification loop [40]. Thus, the intracellular effect of compound **1** on caspase-8 activity, as representative for the extrinsic apoptosis signaling pathways, was examined and results were expressed as percentage of apoptotic cells with active caspase-8 (Figure 5C). Compound **1** at the highest tested concentration proved to promote a significant activation (26.7%) of caspase-8 in apoptotic cells.

These data reveal that derivative **1** activates both apoptosis signaling pathways, potentially inducing an optimal activation of the apoptotic machinery.

#### 2.2.6. Physicochemical Properties Prediction

Taking curcumin burdens into account, and in order to preliminarily exclude the possibility that the synthetized derivatives could act as promiscuous bioactive molecules [41], compounds **1**–**6** were analyzed for their assay interference potential according to three different in silico tools: FAFDrugs4 (http://fafdrugs4.mti.univ-paris-diderot.fr/) [42], False Positive Remover (http://www.cbligand.org/PAINS/) [43] and Aggregator Advisor. (http://advisor.bkslab.org/) [44]. All derivatives were not recognized as potential PAINS or aggregators (Tables S1–S3, Supplementary Materials).

#### 2.2.7. FACS analysis to Study PAINS-Like Behavior

Autofluorescence is one cause of general assays interference and characterizes at least 3.1% of PAINS [16]. As many biologic assays exploit fluorescent probes, autofluorescence is one of the trickiest aspects that mislead about the activity of a compound. False-positive or -negatives are the severe consequences. Aware of this, and since flow cytometry was used to perform all the biologic experiments, compounds’ autofluorescence was taken into account and dealt with.

First, we had to get the lay of the land. In the FACS, unstained cell samples (i.e., with no additional probe) treated with the same concentrations of compounds and time points used for the real biologic assays were run. Two different lasers 488 nm (blue) or 642 nm (far-red), were employed to excite samples. Empirically, it was remarked that all unstained compounds emit fluorescence only if excited with the blue laser. After excitation with this laser, light was filtered and assigned into three emission channels: green, yellow and red. Cells treated with compounds **1**–**6** showed high green fluorescence intensity and low yellow and red ones. With this in mind and since each probe has specific characteristics such as brightness and intensity, for each assay the setting had to be adjusted, in terms of voltage and the signal was compensated in order to cancel the autofluorescence of the analyzed compound.

As expected, the green channel was the most laborious to manage and it was adjusted for caspase-8 activity detection since the caspase-8 substrate emits there. Specifically, with the Guava easyCyte 6HT-2L, it was not possible lower autofluorescence of compound **1** enough to allow performing the experiment. To cope with this situation, the more performant BD FACS Canto cytometer was employed. Figure S7 (Supplementary Materials) shows the gating strategy for that analysis and the successful management of green autofluorescence of **1**. Furthermore, a similar, very recent methodologic approach was applied by Sala de Oyanguren and coworkers, who successfully managed the relevant green autofluorescence of curcumin and the specific signal of a green-emitting dye, as YO-PRO-1 [45]. The interference between any other probe and the compounds’ autofluorescence was handled in the same way. The only exception was that samples were recorded with the FACS Guava. For instance, for the proliferation assay, the red-emitting dye 7-AAD was used. Compounds’ red-autofluorescence was easily displaced and did not interfere with the 7-AAD ones. Over and above, to be sure that artifacts were not recorded, in parallel, FACS results were compared with those obtained by the eosin exclusion dye without observing any significant discordance (data not shown). Conducting cell-cycle analysis was even easier. Indeed, after cell fixation and permeabilization, autofluorescence of derivative **1** was utterly gone (data not shown). Thus, no special adjust setting or compensation was necessary. Last, for apoptosis analysis, 7-AAD and AV–phycoerythrin, which emits in the yellow channel, were used. To combine the phycoerythrin signal to those of 7-AAD and compound **1** resulted in perfectly manageable compensation analysis. All probes used for the remaining biologic assays were excited by 642 nm laser, so we did not experience any trouble. In conclusion, taking into considerations all these aspects we avoided to produce false results and provided solid data demonstrating that as far as autofluorescence is concerned, these derivatives do not behave like PAINS.

#### 2.2.8. Chemical Stability Study

Conscious of the chemical stability liability that may affect the curcumin scaffold, the chemical stability of **1** as mixture of β-keto-enol form and its diketo tautomeric counterpart were studied by RP-HPLC. In detail, a previously reported methodology was applied, and in the working conditions, no degradation products formation was observed (Figure S8, Supplementary Materials).

## 3. Discussion

Herein we reported the design, synthesis and biologic evaluation of a small library of curcumin-triazole conjugates. The conjugation of the “privileged” curcumin framework and triazole-based *para*-fluorine or *para*-methoxy benzyl moieties, allowed obtaining analogs **1**–**6** that were analyzed for their cell growth inhibitory effects on T acute lymphoblastic leukemia cells. The data showed that compounds **1** and **2**, both characterized by the unmodified curcumin–scaffold, significantly inhibited cancer cell line growth with IC_50_ values in the low-μM range. The potency of **1**, proved to be more than three times higher than that of its precursor curcumin, and this compound was selected for deeper evaluation in order to assess the molecular basis of its anticancer profile. The induction of apoptotic process turned out to be the molecular mode by which compound **1** induced cell death. Its peculiar ability to affect both the intrinsic and the extrinsic pathways represents a significant added value, useful to achieve a strengthening in anticancer outcome in terms of cytotoxic response and decreased predisposition of chemoresistance insurgence. However, this evidence would be worthless if the compound under assessment is a PAIN, in other words if the favorable results are just artefacts. Chelation, covalent modifications, redox effect, and autofluorescence are only some of the conditions that push drug candidates into the PAINS qualification [16]. In this study, we focused our attention on the autofluorescence of the derivatives and we efficiently prevented its interference with the biologic assays. We analyzed just one part of the “PAIN puzzle” and our results suggest, but do not prove, that compound **1** is not a PAIN. Certainly, our data represent an interesting premise to keep on exploring antitumor potential of **1** while keeping our eyes wide open.

## 4. Materials and Methods

### 4.1. Chemistry

Chemical reagents and solvents, unless otherwise specified, were employed as commercial products with a high-grade purity. Reaction courses were monitored by thin-layer chromatography (TLC) performed on precoated TLC plates (Silica Gel 60 F254, layer 0.20 mm, Merck, Darmstadt, Germany) and then visualized under a UV lamp (λ = 254 and 365 nm). Chromatographic separations were performed on silica gel columns by using the flash method (flash column chromatography (FCC), Kiesegel 40, particle size 0.040–0.063 mm, Merck, Darmstadt, Germany). Melting points were determined in open glass capillaries, using a Büchi apparatus (Büchi Italia s.r.l., Cornaredo, MI, Italy) and are uncorrected. ^1^H NMR and ^13^C NMR spectra were recorded on Varian INOVA spectrometer (Scientific instruments, Palo Alto, CA, USA) operating at 400 MHz and 101 MHz, respectively; data are reported as follows: chemical shift (ppm δ value), multiplicity (indicated as: br., broad signal; s, singlet; d, doublet; t, triplet; q, quartet; p, quintet; m, multiplet and combinations thereof), coupling constants (*J*) in Hertz (Hz) and integrated intensity. Mass spectra were recorded on a Waters ZQ 4000 apparatus (Waters Alliance, San Diego, CA, USA) operating in electrospray mode (ES). All tested compounds were found to have >95% purity, as determined by HPLC analysis, performed on a chromatograph PU-1587 UV model equipped with a 20 μL loop valve (Jasco Europe, Italy) by using a Phenomenex Luna 5 µm C18 column (150 × 4.60 mm) as stationary phase and a mixture of H_2_O/MeCN (50:50, *v*/*v*) for **1** and **2**, H_2_O/MeCN (35:65, *v*/*v*) for **3**–**6** as mobile phase; detection at λ = 254 nm, flow rate of 1.0 mL/min. Compounds name is in accord with the naming algorithm developed by CambridgeSoft Corporation used in Chem-BioDraw Ultra 19.1 (Perkin Elmer, Waltham, MA, USA.

#### 4.1.1. Huisgen 1,3-Dipolar Cycloaddition. General Procedure for the Synthesis of Curcumin-Triazole Conjugates **1**, **2** and Intermediates **13**, **14**

To a solution of the corresponding alkyne **8** or **12** (1.00 mmol) and azide **9** or **10** (1.3 molar equiv) in DMF (7.50 mL), TEA (0.1 molar equiv) was added dropwise, followed by a water solution of CuSO_4_ (0.1 molar equiv) and (+)-sodium *L*-ascorbate (0.5 molar equiv). The resulting mixture was stirred for 2–18 h at rt and the reaction progress was monitored by TLC. Upon reaction completion, the solution was poured into H_2_O and the formed solid was filtered off and dried under vacuum.

(1*E*,4*Z*,6*E*)-4-((1-(4-fluorobenzyl)-1H-1,2,3-triazol-4-yl)methyl)-5-hydroxy-1,7-bis(4-hydroxy-3-methoxyphenyl)hepta-1,4,6-trien-3-one (**1-KE**); (1*E*,6*E*)-4-((1-(4-fluorobenzyl)-1H-1,2,3-triazol-4-yl)methyl)-1,7-bis(4-hydroxy-3-methoxyphenyl)hepta-1,6-diene-3,5-dione (**1-KK**).

According to the general procedure, **8-KE/KK** (0.24 g, 0.60 mmol) and **9** (0.12 g, 0.78 mmol) were allowed to react for 18 h. The crude product was purified by FCC on silica gel (EP/EtOAc 1:1) and further crystallized from DCM/EP to obtain **1-KE/KK** as a red solid (0.18 g, 1.0:1.1 mixture ratio of KE/KK tautomer), 41% yield, mp 95–97 °C. ^1^H NMR (CDCl_3_) δ 3.38 (d, *J* = 7.2 Hz, 2H, CH_2_, KK), 3.91 (s, 12H, OCH_3_, KE+KK), 4.06 (s, 2H, CH_2_, KE), 4.76 (t, *J* = 7.2 Hz, 1H, CH, KK), 5.40 (s, 4H, CH_2_ N, KE+KK), 5.92 (br, 2H, OH, KE), 5.98 (br, 2H, OH, KK), 6.67 (d, *J* = 16.0 Hz, 2H, CH=CH, KK), 6.81–6.88 (m, 4H, Ar and CH=CH, KE), 6.91 (d, *J* = 8.0 Hz, 4H, H-5, KK+KE), 6.93–6.99 (m, 4H, Ar and H-2, KE), 7.01 (s, 2H, H-2, KK), 7.03–7.11 (m, 6H, Ar and H-6, KK+KE), 7.14–7.18 (m, 2H, Ar), 7.26 (s, 2H, CH-triazole, KE+KK), 7.58 (d, *J* = 15.6 Hz, 2H, CH=CH, KK), 7.67 (d, *J* = 15.2 Hz, 2H, CH=CH, KE). ^13^C NMR (CDCl_3_) δ 23.4 (CH_2_, KE), 24.9 (CH_2_, KK), 53.4 (CH_2_ N), 53.6 (CH_2_ N), 56.2 (4C, OCH_3_), 63.1 (CH), 77.4, 107.8 (2C, CH), 109.8 (4C, CH), 115.0 (4C, CH), 116.2 (d, *J* = 21.8 Hz, 4C, CH), 117.9 (2C, CH=CH, KE), 122.0 (2C), 122.1 (2C, CH=CH, KK), 122.2, 123.4 (2C, CH), 124.3 (2C, CH), 126.8 (2C), 127.9 (2C), 129.7 (d, *J* = 8.4 Hz, 2C, CH), 129.9 (d, *J* = 8.4 Hz, 2C, CH), 130.6 (d, *J* = 3.2 Hz), 130.7 (d, *J* = 3.2 Hz), 142.5 (2C, CH=CH, KE), 145.3 (2C, CH=CH, KK), 145.4 (2C), 148.2 (d, *J* = 255.6 Hz, 2C), 147.0 (2C), 148.3 (2C), 148.9 (2C), 183.3 (2C), 194.6 (2C). ESI-MS (*m*/*z*) 580 (M + Na).

The same reaction was performed starting from **8-KE** and **1-KE/KK** was obtained as 1.3:1.0 mixture ratio of KE/KK tautomers.

(1*E*,4*Z*,6*E*)-5-hydroxy-1,7-bis(4-hydroxy-3-methoxyphenyl)-4-((1-(4-methoxybenzyl)-1*H*-1,2,3-triazol-4-yl)methyl)hepta-1,4,6-trien-3-one (**2-KE**); (1*E*,6*E*)-1,7-bis(4-hydroxy-3-methoxyphenyl)-4-((1-(4-methoxybenzyl)-1*H*-1,2,3-triazol-4-yl)methyl)hepta-1,6-diene-3,5-dione (**2-KK**).

Following the general procedure, **8-KE/KK** (0.36 g, 0.89 mmol) and **10** (0.19 g, 1.15 mmol) were allowed to react for 18 h. Purification by FCC (EP/EtOAc 7:3) and further crystallization from DCM/EP gave **2-KK/KE** as a red solid (0.22 g, 1.0:1.3 mixture ratio of KE/KK tautomer), 43% yield, mp 98–100 °C. ^1^H NMR (CDCl_3_) δ 3.35 (d, *J* = 7.2 Hz, 2H, CH_2_, KK), 3.68 (s, 3H, OCH_3_, KE), 3.72 (s, 3H, OCH_3_, KK), 3.90 (s, 12H, OCH_3_, KE+KK), 4.03 (s, 2H, CH_2_, KE), 4.74 (t, *J* = 7.2 Hz, 1H, CH, KK), 5.35 (s, 4H, CH_2_ N, KE+KK), 5.88 (br, 2H, OH, KK), 5.92 (br, 2H, OH, KE), 6.65 (d, *J* = 6.8 Hz, 4H, H-5, KE+KK), 6.68 (s, 4H, H-2, KE+KK), 6.78 (d, *J* = 8.8 Hz, 4H, H-6, KE+KK), 6.83 (d, *J* = 15.2 Hz, CH=CH, 2H, KK), 6.89 (d, *J* = 8.0 Hz, 4H, Ar, KE+KK), 6.95 (s, 1H, CH-triazole, KE), 7.00 (s, 1H, CH-triazole, KK), 7.04 (d, *J* = 8.4 Hz, 4H, Ar, KE+KK), 7.11 (d, *J* = 15.2 Hz, 2H, CH=CH, KE), 7.56 (d, *J* = 15.6 Hz, 2H, CH=CH, KK), 7.66 (d, *J* = 15.6 Hz, 2H, CH=CH, KE). ^13^C NMR (CDCl_3_) δ 23.4, 24.8, 29.8, 53.7, 53.9, 55.3, 55.4, 56.1 (2C), 56.2 (2C), 62.9, 107.8 (2C), 109.8 (2C), 109.9 (2C), 114.4 (2C), 114.5 (2C), 114.9 (2C), 115.0 (2C), 117.9, 121.9, 122.1 (2C), 123.5 (2C), 124.3 (2C), 127.6 (2C), 127.9 (2C), 129.3 (2C), 129.6 (2C), 129.7, 142.5, 145.1 (2C), 145.3 (2C), 147.0 (2C), 148.3 (2C), 149.0 (2C), 149.2 (2C), 159.9 (2C), 183.2 (2C), 194.6 (2C). ESI-MS (*m*/*z*): 592 (M + Na).

The same reaction was performed starting from **8-KE** and **2-KE/KK** was obtained as 1.0:1.2 mixture ratio of KE/KK tautomers.

4-((1-(4-fluorobenzyl)-1*H*-1,2,3-triazol-4-yl)methoxy)benzaldehyde (**13**).

In line with the general procedure, **12** (0.19 g, 1.20 mmol) and **9** (0.24 g, 1.55 mmol) were allowed to react for 2 h. The formed solid was collected by vacuum filtration to afford **13** (0.16 g) as pure solid, 43% yield, mp 88–90 °C. ^1^H NMR (CDCl_3_) δ 5.25 (s, 2H, OCH_2_), 5.51 (s, 2H, CH_2_ N), 7.04–7.09 (m, 4H, Ar), 7.28 (d, *J* = 8.4 Hz, 2H, Ar), 7.51 (s, 1H, CH-triazole), 7.82 (d, *J* = 8.4 Hz, 2H, Ar), 9.88 (s, 1H, CHO).

4-((1-(4-methoxybenzyl)-1*H*-1,2,3-triazol-4-yl)methoxy)benzaldehyde (**14**).

In line with the general procedure, **12** (0.21 g, 1.28 mmol) and **10** (0.27 g, 1.67 mmol) were allowed to react for 3 h. The formed solid was collected by vacuum filtration to afford **14** (0.25 g) as pure solid, 60% yield, mp 83–85 °C. ^1^H NMR (CDCl_3_) δ 3.81 (s, 3H, OCH_3_), 5.25 (s, 2H, OCH_2_), 5.48 (s, 2H, CH_2_ N), 6.90 (d, *J* = 8.4 Hz, 2H, Ar), 7.08 (d, *J* = 8.4 Hz, 2H, Ar), 7.24 (d, *J* = 8.4 Hz, 2H, Ar), 7.51 (s, 1H, CH-triazole), 7.83 (d, *J* = 8.4 Hz, 2H, Ar), 9.89 (s, 1H, CHO).

#### 4.1.2. Pabon Reaction. General Procedure for the Synthesis of Curcumin-Triazole Conjugates **3**–**6** and Intermediates **8** and **11**

A suspension of 2,4-dicarbonyl compound (pentane-2,4-dione, intermediates **7** or **11** [20], 1.00 mmol) and B_2_O_3_ (1.0 molar equiv) in DMF (1.50 mL), was heated for 30 min at 80 °C, then B(*n*-BuO)_3_ (2.0 molar equiv for **3**, **4** and **11**, or 4.0 molar equiv for **5**, **6** and **8** derivatives) was added and the resulting mixture was heated at the same temperature for additional 30 min. Afterward, a solution of the suitable aldehyde/s, (0.9 molar equiv for **3**, **4** and **11** or 1.8 molar equiv for **5**, **6** and **8**) in DMF (1.00 mL) was added, followed by a *n*-BuNH_2_ solution (0.2 molar equiv for **3**, **4** and **11**, or 0.4 molar equiv for **5**, **6** and **8**, in 1.00 mL of DMF). After stirring at 80 °C for 6–8 h, the resulting mixture was cooled to rt, acidified with HCl (0.5 N, 8.0 mL) and stirred at 80 °C for further 30 min. On cooling, a solid product precipitates, which was separated by vacuum filtration. The obtained crude was suspended in H_2_O, filtered and purified by FCC and/or crystallization from suitable solvent or mixture of solvents, to afford the title pure compound.

(1*E*,4*Z*,6*E*)-1-(4-((1-(4-Fluorobenzyl)-1*H*-1,2,3-triazol-4-yl)methoxy)phenyl)-5-hydroxy-7-(4-hydroxy-3-methoxyphenyl)hepta-1,4,6-trien-3-one (**3**).

Following the general procedure, reaction of intermediate **11** (0.07 g, 0.29 mmol) and the aldehyde **13** (0.08 g, 0.26 mmol) gave the crude which was purified by FCC (PE/EtOAc 6:4) and further crystallization from EtOH to obtain **3** as an orange powder (0.05 g), 40% yield, mp 113–115 °C. ^1^H NMR (CDCl_3_) δ 3.95 (s, 3H, OCH_3_), 5.23 (s, 2H, CH_2_ N), 5.52 (s, 2H, OCH_2_), 5.80 (s, 1H, CH), 6.49 (d, *J* = 15.6 Hz, 1H, CH=CH), 6.50 (d, *J* = 15.6 Hz, 1H, CH=CH), 6.94 (d, *J* = 8.4 Hz, 1H, H-5), 6.99 (d, *J =* 7.6 Hz, 2H, Ar), 7.06 (s, 1H, H-2), 7.07 (d, *J* = 7.6 Hz, 1H, H-6), 7.08–7.14 (m, 2H, Ar), 7.24–7.27 (m, 2H, Ar), 7.50 (d, *J* = 8.0 Hz, 2H, Ar), 7.55 (s, 1H, CH-triazole), 7.60 (d, *J* = 15.6 Hz, 1H, CH=CH), 7.61 (d, *J* = 16.0 Hz, 1H, CH=CH). ^13^C NMR (CDCl_3_) δ 53.7, 56.1, 62.2, 101.5, 109.7, 114.9, 115.3 (2C), 116.4 (d, *J* = 21.4 Hz, 2C), 121.9, 122.3, 122.7, 123.1, 127.8, 128.5, 129.9 (2C), 130.0, 130.2 (d, *J* = 8.1 Hz, 2C), 140.0, 140.8, 144.4, 146.9, 148.0, 159.9, 160.7 (d, *J* = 249.4 Hz), 183.2, 183.6. ESI-MS (*m/z*): 550 (M + Na).

(1*E*,4*Z*,6*E*)-5-hydroxy-7-(4-hydroxy-3-methoxyphenyl)-1-(4-((1-(4-methoxybenzyl)-1*H*-1,2,3-triazol-4-yl)methoxy)phenyl)hepta-1,4,6-trien-3-one (**4**).

According to the general procedure, reaction of intermediate **11** (0.20 g, 0.86 mmol) and the aldehyde **14** (0.25 g, 0.77 mmol) gave the crude which was purified by FCC (PE/EtOAc 6:4) and further crystallization from EtOH to give **4** as an orange-brown powder (0.19 g), 45% yield, mp 149–151 °C. ^1^H NMR (CDCl_3_) δ 3.82 (s, 3H, OCH_3_), 3.96 (s, 3H, OCH_3_), 5.22 (s, 2H, OCH_2_), 5.48 (s, 2H, NCH_2_), 5.80 (s, 1H, CH), 5.88 (br, 1H, OH), 6.49 (d, *J* = 15.6 Hz, 1H, CH=CH), 6.50 (d, *J* = 16.0 Hz, 1H, CH=CH), 6.91 (d, *J* = 8.4 Hz, 2H, Ar), 6.94 (d, *J* = 8.0 Hz, 1H, H-5), 6.99 (d, *J* = 8.8 Hz, 2H, Ar), 7.06 (d, *J* = 1.6 Hz, 1H, H-2), 7.13 (dd, *J* = 1.6 and 8.4 Hz, 1H, H-6), 7.25 (d, *J* = 8.8 Hz, 2H, Ar), 7.50 (d, *J* = 8.8 Hz, 2H, Ar), 7.51 (s, 1H, CH-triazole), 7.60 (d, *J* = 16.0 Hz, 1H, CH=CH), 7.61 (d, *J* = 16.0 Hz, 1H, CH=CH). ^13^C NMR (CDCl_3_) δ 53.9, 55.5, 56.1, 62.2, 101.5, 109.7, 114.6 (2C), 115.0, 115.3 (2C), 121.7, 122.2, 122.6, 123.1, 126.4, 127.8, 128.4, 129.9 (4C), 140.0, 140.7, 144.1, 147.0, 148.0, 159.9, 160.1, 183.2, 183.6. ESI-MS (*m*/*z*): 538 (M—H).

(1*E*,4*Z*,6*E*)-1,7-bis(4-((1-(4-fluorobenzyl)-1*H*-1,2,3-triazol-4-yl)methoxy)phenyl)-5-hydroxyhepta-1,4,6-trien-3-one (**5**).

In line with the general procedure, reaction of pentane-2,4-dione (0.03 mL, 0.29 mmol) and the aldehyde **13** (0.16 g, 0.52 mmol) gave the crude product which was purified by FCC (PE/EtOAc 1:1) and further crystallization from DCM/PE to afford **5** as a yellow powder (0.07 g), 35% yield, mp 134–136 °C. ^1^H NMR (CDCl_3_) δ 5.22 (s, 4H, OCH_2_), 5.52 (s, 4H, CH_2_ N), 5.79 (s, 1H, CH), 6.51 (d, *J* = 15.6 Hz, 2H, CH=CH), 7.00 (d, *J* = 8.8 Hz, 4H, Ar), 7.06–7.10 (m, 4H, Ar), 7.27–7.30 (m, 4H, Ar), 7.51 (d, *J* = 8.4 Hz, 4H, Ar), 7.54 (s, 2H, CH-triazole), 7.62 (d, *J* = 15.6 Hz, 2H, CH=CH). ^13^C NMR (CDCl_3_): δ 53.7 (2C), 62.2 (2C), 101.5, 115.3 (4C), 116.35 (d, *J* = 21.4 Hz, 4C), 121.9, 122.3, 122.7 (2C), 128.5 (2C), 129.9 (4C), 130.0 (2C), 130.2 (d, *J* = 8.1 Hz, 4C), 140.0, 140.8, 144.4 (2C), 160.0 (2C), 160.7 (d, *J* = 249.4 Hz, 2C), 183.2, 183.6. ESI-MS (*m/z*): 687 (M + H).

(1*E*,4*Z*,6*E*)-5-hydroxy-1,7-bis(4-((1-(4-methoxybenzyl)-1*H*-1,2,3-triazol-4-yl)methoxy)phenyl)hepta-1,4,6-trien-3-one (**6**).

Following the general procedure, reaction of pentane-2,4-dione (0.09 mL, 0.86 mmol) and the aldehyde **14** (0.50 g, 1.55 mmol) gave the crude product which was purified by FCC (PE/EtOAc 1:1) affording **6** as an orange-brown powder (0.18 g), 30% yield, mp 173–175 °C. ^1^H NMR (CDCl_3_) δ 3.82 (s, 6H, OCH_3_), 5.22 (s, 4H, OCH_2_), 5.48 (s, 4H, CH_2_ N), 5.79 (s, 1H, CH), 6.48 (d, *J* = 15.6 Hz, 2H, CH=CH), 6.88 (d, *J* = 8.0 Hz, 4H, Ar), 6.97 (d, *J* = 8.0 Hz, 4H, Ar), 7.25 (d, *J* = 8.0 Hz, 4H, Ar), 7.48 (d, *J* = 8.0 Hz, 4H, Ar), 7.48 (s, 2H, CH-triazole), 7.59 (d, *J* = 15.6 Hz, 2H, CH=CH). ^13^C NMR (CDCl_3_) δ 54.0 (2C), 55.5 (2C), 62.3 (2C), 101.6, 114.7 (4C), 115.4 (4C), 122.3 (2C), 122.6 (2C), 126.5 (2C), 128.5 (2C), 129.9 (8C), 134.7, 140.1, 144.2 (2C), 160.2 (2C), 167.3 (2C), 183.4 (2C). ESI-MS (*m*/*z*): 733 (M + Na).

(1*E*,4*Z*,6*E*)-5-hydroxy-1,7-bis(4-hydroxy-3-methoxyphenyl)-4-(prop-2-yn-1-yl)hepta-1,4,6-trien-3-one (**8-KE**); (1*E*,6*E*)-1,7-bis(4-hydroxy-3-methoxyphenyl)-4-(prop-2-yn-1-yl)hepta-1,6-diene-3,5-dione (**8-KK**).

Following the general procedure, reaction of **7** (0.36 g, 2.61 mmol) and 3-methoxy-4-hydroxybenzaldehyde (0.71 g, 4.70 mmol) gave a crude product which was purified by FCC (PE/EtOAc 7:3) and further crystallization from EtOH. In this case, the desired compound was isolated as **8-KE** pure and as 1.0:1.7 mixture of **8-KK/KE** tautomers (0.61 g), 58% yield, mp 144–147 °C. **8-KE**: ^1^H NMR (CDCl_3_) δ 2.15 (t, *J* = 2.4 Hz, 1H, C≡CH), 3.44 (d, *J* = 2.4 Hz, 2H, CH_2_C≡), 3.96 (s, 6H, OCH_3_), 5.89 (br, 2H, OH), 6.95 (d, *J* = 8.0 Hz, 2H,H-5), 6.99 (d, *J* = 15.4 Hz, 2H, CH=CH), 7.07 (d, *J* = 2.0 Hz, 2H, H-2), 7.20 (dd, *J* = 8.0 and 2.0 Hz, 2H, H-6), 7.73 (d, *J* = 15.4 Hz, 2H, CH=CH). **8-KK/KE**: ^1^H NMR (CDCl_3_) δ 2.15 (t, *J* = 2.4 Hz, 2H, C≡CH, KK+KE), 2.91 (dd, *J* = 7.4 and 2.4 Hz, 2H, CH_2_C≡, KK), 3.44 (d, *J* = 2.4 Hz, 2H, CH_2_C≡, KE), 3.96 (s, 12H, OCH_3_, KK+KE), 4.34 (t, *J* = 7.4 Hz, 1H, CH, KK), 5.89 (br, 2H, OH, KE), 5.94 (br, 2H, OH, KK), 6.70 (d, *J* = 15.4 Hz, 2H, CH=CH, KK), 6.91 (d, *J* = 8.0 Hz, 2H,H-5, KK), 6.95 (d, *J* = 8.0 Hz, 2H,H-5, KE), 6.99 (d, *J* = 15.4 Hz, 2H, CH=CH, KE), 7.04 (d, *J* = 2.0 Hz, 2H, H-2, KK), 7.07 (d, *J* = 2.0 Hz, 2H, H-2, KE), 7.12 (dd, *J* = 8.0 and 2.0 Hz, 2H, H-6, KK), 7.20 (dd, *J* = 8.0 and 2.0 Hz, 2H, H-6, KE), 7.66 (d, *J* = 15.4 Hz, 2H, CH=CH, KK), 7.73 (d, *J* = 15.4 Hz, 2H, CH=CH, KE).

3*Z*,5*E*-4-hydroxy-6-(4-hydroxy-3-methoxyphenyl)hexa-3,5-dien-2-one (**11**) [20].

In line with the general procedure, reaction of pentane-2,4-dione (1.00 g, 10.00 mmol) and vanillin (1.37 g, 9.00 mmol) gave a crude product which was purified by FCC (PE/EtOAc 9.75:0.25), affording **11** as a yellow solid (1.16 g), 55% yield, mp 144–146 °C. ^1^H-NMR (CDCl_3_) δ 2.16 (s, 3H, CH_3_), 3.94 (s, 3H, OCH_3_), 5.40 (br, 1H, OH), 5.63 (s, 1H, CH), 6.33 (d, *J* = 16.0 Hz, 1H, CH=CH), 6.92 (d, *J* = 8.0 Hz, 1H, H-5), 7.02 (d, *J* = 1.8 Hz, 1H, H-2), 7.09 (dd, *J* = 8.0 and 1.8 Hz, 1H, H-6), 7.53 (d, *J* = 16.0 Hz, 1H, CH=CH). The spectroscopic data of this intermediate are in good agreement with those reported in the literature.

General Procedure for the Synthesis of Azido Intermediates **9** and **10**.

A mixture of the appropriate benzyl halide (1.00 mmol) and NaN_3_ (10.0 molar equiv) in DMF (10.0 mL) was stirred at 60 °C for 10 h. After cooling to rt the reaction mixture was poured into H_2_O and extracted with Et_2_O (3 × 20.0 mL); the combined organic layers were washed with brine, dried over Na_2_SO_4_ and concentrated to dryness. The desired azide was employed in the next synthetic step without any further purification.

1-(azidomethyl)-4-fluorobenzene (**9**)

Following the general procedure and starting from 4-fluorobenzyl bromide (0.66 mL, 5.29 mmol) and NaN_3_ (3.44 g, 52.90 mmol), the title compound **9** was obtained as a yellow oil, 50% yield. ^1^H NMR (CDCl_3_) δ 4.29 (s, 2H, CH_2_ N_3_), 7.03–7.07 (m, 2H, Ar), 7.24–7.29 (m, 2H, Ar).

1-(azidomethyl)-4-methoxybenzene (**10**)

Following the general procedure and starting from 4-methoxybenzyl chloride (0.86 mL, 6.38 mmol) and NaN_3_ (4.15 g, 63.80 mmol), the title compound **10** was obtained as a yellow oil, 67% yield. ^1^H NMR (CDCl_3_) δ 3.82 (s, 3H, OCH_3_), 4.28 (s, 2H, CH_2_ N_3_), 6.92 (d, *J* = 8.4 Hz, 2H, Ar), 7.26 (d, *J* = 8.4 Hz, 2H, Ar).

4-(Prop-2-yn-1-yloxy)benzaldehyde (**12**) [46]

To a stirred suspension of 4-hydroxybenzaldehyde (1.00 g, 8.19 mmol) and anhydrous Κ_2_CO_3_ (1.36 g, 9.83 mmol) in acetone (100 mL), propargyl bromide solution (80 wt% in toluene, 1.09 mL, 9.83 mmol) was added dropwise. The reaction mixture was stirred under reflux for 8 h, monitoring the reaction progress by TLC. The mixture was hot filtered, and the solvent was evaporated under reduced pressure. The resulting crude product was purified by FCC (PE/EtOAc 4:1) to afford **12** as a pale yellow solid, 78% yield, mp 75−77 °C. ^1^H-NMR (CDCl_3_) δ 2.81 (t, *J* = 2.4 Hz, 1H, ≡CH), 4.96 (d, *J* = 2.4 Hz, 2H, CH_2_C≡), 7.10 (d, 2H, *J* = 7.6 Hz, Ar), 7.83 (d, 2H, *J* = 7.6 Hz, Ar), 9.90 (s, 1H, CHO). The spectroscopic data of this intermediate are in good agreement with those reported in the literature.

(*Z*)-3-(1-hydroxyethylidene)hex-5-yn-2-one (**7-KE**); 3-(prop-2-yn-1-yl)pentane-2,4-dione (**7-KK**).

A stirred suspension of pentane-2,4-dione (2.59 mL, 25.17 mmol), propargyl bromide solution (80 wt% in toluene, 1.87 mL, 16.86 mmol), Κ_2_CO_3_ (2.32 g, 16.86 mmol) in acetone (150 mL) was heated at 80 °C for 6 h, monitoring the reaction progress by TLC. The mixture was hot filtered, and the solvent was evaporated under reduced pressure. The resulting crude was purified by FCC (PE/DCM 4:1) to isolate the desired compound as **7-KE** pure and as 1.7:1.0 mixture of **7-KK/KE** tautomers (1.40 g), 60% yield, mp 42–45 °C. **7-KE**: ^1^H NMR (CDCl_3_) δ 2.03 (t, *J* = 2.4 Hz, 1H, C≡CH), 2.18 (s, 6H, CH_3_), 2.99 (d, *J* = 2.4 Hz, 2H, CH_2_C≡). **7-KK/KE**: ^1^H NMR (CDCl_3_) δ 2.03 (t, *J* = 2.4 Hz, 2H, C≡CH, KK+KE), 2.18 (s, 6H, CH_3_, KE), 2.26 (s, 6H, CH_3_, KK), 2.70 (dd, *J* = 2.8 and 7.2 Hz, 2H, CH_2_C≡, KK), 2.99 (d, *J* = 2.4 Hz, 2H, CH_2_C≡, KE), 3.86 (t, *J* = 7.2 Hz, 1H, CH).

### 4.2. Cell Cultures and Treatments

Human CCRF–CEM acute lymphoblastic leukemia T cells and Jurkat acute T leukemia cells were purchased from ATCC (Manassas, VA, USA) and propagated in RPMI 1640 (ATCC), supplemented with 10% heat-inactivated fetal bovine serum (FBS, Biochrome, a division of Harvard Bioscience, Inc., Holliston, MA, USA), 1% L-glutamine (Sigma-Aldrich, Saint Louis, MO, USA) and 1% penicillin/streptomycin solution 100 U/mL (Sigma-Aldrich, Saint Louis, MO, USA). Cells were maintained at 37 °C in a humidified atmosphere containing 5% CO_2_. For all experiments, to keep cells in their logarithmic growth phase, they were seeded at a density of 0.25 × 10^6^ cells/mL and treated with increasing concentrations of the different compounds for different time points, depending on the assay.

### 4.3. Analysis of Cell Viability

CCRF–CEM was treated with increasing concentrations of all compounds or the reference compound curcumin for 24 and 48 h and analyzed with the Guava ViaCount Reagent (Merck KGaA, Darmstadt, Germany), following the manufacturer instructions. Briefly, cells were diluted with the reagent containing 7-AAD and incubated at room temperature in the dark for 5 min before been recorded at the flow cytometer. Then, to confirm the solidity of these data, viability was also determined by the eosin exclusion dye. Viable cells were counted using a Bürker chamber and light microscopy (Nikon eclipse TS2, Amsterdam, Netherlands). The IC_50_ was calculated by interpolation from the nonlinear dose–response curve.

Cell death analysis with or without specific inhibitors on Jurkat cells was performed as follow. Briefly, cells were pretreated for 1 h with or without the pan-caspase inhibitor carbobenzoxy-valyl-alanyl-aspartyl-[O-methyl]-fluoromethylketone (zVAD-fmk, 10 μM, Bachem, Bubendorf, Switzerland), the RIPK1 inhibitor necrostatin-1 s (Nec1s, 10 μM, Abcam, Cambridge, UK), the inhibitor of ROS and lipid peroxidation ferrostatin-1 (Fer-1, 1 μM, Sigma-Aldrich, St. Louis, MO, USA) or the iron chelator deferoxamine (DFO, 10 μM, Sigma-Aldrich) to, respectively block apoptosis, necroptosis or ferroptosis. Then, Jurkat were treated for 48 h with compound **1** 6 µM and cell death was analyzed through the fluorescent apoptosis/necrosis (FAN) assay [37] using the cell-impermeant fluorescent nuclear probe Sytox Red (Thermo Fisher Scientific, Waltham, MA, USA). Fluorescence was measured on a Tecan Spark^®^ 20-M multimode microplate reader.

### 4.4. Analysis of Cell Cycle

After treatment with **1** for 24 h, cells were fixed with 70% ice-cold ethanol and, after washing, suspended in 200 μL of Guava cell cycle reagent (Merck KGaA), containing propidium iodide. At the end of incubation at room temperature for 30 min in the dark, samples were analyzed via flow cytometry.

### 4.5. Analysis of Cell Death

After 24 h treatments with **1**, aliquots of 2 × 10^4^ CCRF–CEM were resuspended in 100 µL of RPMI 1640 containing at least 10% of FBS and stained with an equal volume of Guava Nexin Reagent (Merck KGaA) containing annexin V-phycoerythrin and 7-amino-actinomycin D (7-AAD) and analyzed via flow cytometry.

### 4.6. Analysis of Mitochondrial Transmembrane Potential

The mitochondrial transmembrane potential was assessed using MitoProbeTM DilC1(5) Assay kit (Molecular Probes, Thermo Fisher Scientific, Waltham, MA, USA). Briefly, after 24 h from compound **1** treatment, 10^6^ cells were washed and treated with 50-nM DilC1(5) (1,1′,3,3,3′,3′-hexamethylindo dicarbocyanine iodide) for 20 min at 37 °C, 5% CO_2_. Cells were washed with PBS 1X (Sigma-Aldrich) and suspended for flow cytometric analysis in a solution composed of equal volumes of RPMI 1640 containing at least 10% of FBS and Guava Nexin Reagent. CCCP (carbonyl cyanide 3-chlorophenylhydrazone) was used as positive control.

### 4.7. Evaluation of Caspase-8 Activity

The activity of caspase-8 cells was assessed using the CaspGLOW Fluorescein Active Caspase 8 Staining Kit (Invitrogen, Thermo Fisher Scientific, Waltham, MA, USA). Briefly, after 24 h from compound **1** treatment, 106 cells were washed and incubated for 30 min with the reagent. Cells were washed again, stained with 7-AAD (BD Bioscience, Franklin Lakes, NJ, USA) for 1 h and read flow cytometrically. Results were expressed as percentage of apoptotic cells with active caspase 8. Apoptotic cells were identified thanks to the staining with 7-AAD (Supplementary Figure S7D) [47].

On note, compound **1** green autofluorescence was recorded during this assay, but did not affect the analysis. Gating strategy and autofluorescence handling are showed in Figure S7, Supplementary Materials.

### 4.8. Flow Cytometry

All procedures except for the analysis of caspase 8 activity were performed with a Guava EasyCyte 6HT-2 L (Merck KGaA) while for caspase 8 FACSCanto cytometer (Becton Dickinson, BD, San Jose, CA, USA) was used. At least 10,000 events (cells) were recorded for each sample. In all cytofluorimetric analyses, cell debris and clumps were gated out.

### 4.9. Statistical Analysis

All biologic experiments were performed at least in triplicates. All results are expressed as mean ± SEM. Differences among treatments were evaluated by repeated ANOVA, followed by Bonferroni or Dunnett as posttest, using GraphPad InStat version 6.00 (GraphPad Prism, San Diego, CA, USA). *p* < 0.05 was considered significant.

### 4.10. Chemical Stability Study

The tested derivative **1** was dissolved in DMSO (0.50 mg/mL) and the pH of the solution was adjusted to 7.4 using 50-mM phosphate. The obtained solutions were then maintained at room temperature and at 70 °C (oven) for 48 h and analyzed by RP-HPLC under the conditions reported in the Section 2.2.8.

## 5. Conclusions

The obtained data reveal that derivative **1** activates both apoptosis signaling pathways, potentially inducing an optimal activation of the apoptotic machinery. This peculiar property offers promises for achieving an amplified anticancer potential. Further studies will be also necessary to draw a toxicological profile of compound **1** in order to elaborate a risk/benefit analysis. Given the high tolerability and low toxicity of curcumin in terms of selectivity for tumor cells and lack of toxicity in vivo [48,49,50], we expect compound **1** to be not less. The complete pharmaco-toxicological profile will support assessing if this compound could be regarded as suitable for its development as an effective anticancer therapeutics. Follow-up studies will be devoted to deeply investigate the molecular mode of its cell death induction. Moreover, an extensive structure–activity relationship (SAR) study is now ongoing focused at a rational optimization of this lead structure. The outcomes of these efforts will be reported in due time.

## Figures and Tables

**Table 1:** Cytotoxic effect of compounds **1**–**6** against T acute lymphoblastic leukemia cell line (CCRF–CEM).

Compound	R	CCRF–CEM Cell Line IC_50_ (µM) ^a^	
		24 h	48 h
**1**	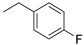	68.30	3.13
**2**	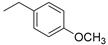	55.63	3.95
**3**	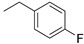	38.68	38.00
**4**	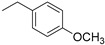	>150	69.07
**5**	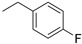	>150	88.94
**6**	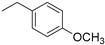	122.1	93.40
**curcumin**	/	>150	10.51

^a^ IC_50_ values were calculated by interpolation from the nonlinear dose–response curve of raw data.

## Supplementary Materials

The following are available online, Figure S1. ^1^H NMR (CDCl_3_, 400 MHz) copy of compound **1**, Figure S2. ^13^C NMR copy of compound **1**, Figure S3. ^1^H–^1^H COSY copy of compound **1**. Figure S4. ^1^H–^13^C HSQC copy of compound **1**, Figure S5. 2D NOESY copy of compound **1**, Figure S6. Cell-death analysis of Jurkat cells pretreated with zVAD-fmk, Nec1s, DFO or Fer-1 and then treated with compound **1** for 48 h, Figure S7. Gating strategy (A, B, C, D) and green autofluorescence management (E) in caspase 8 analysis, Figure S8. HPLC copy for compound **1**; Table S1 and S2. Physicochemical properties prediction for compounds **1**–**6**; Table S3. PAINS filters for compounds **1**–**6**.
