# Supplementary material for: Curcumin-1,2,3-Triazole Conjugation for Targeting the Cancer Apoptosis Machinery
Source: Molecules. 2020 Jul 5;25(13):3066. doi: 10.3390/molecules25133066 (PMC7412087; doi:10.3390/molecules25133066)
Supplement: Supplementary file 1 [file molecules-25-03066-s001.pdf]

Supplementary Materials

Curcumin-1,2,3-Triazole Conjugation for Targeting the Cancer Apoptosis Machinery

Francesca Seghetti[1^], Rita Maria Concetta Di Martino[1^§], Elena Catanzaro[2^], Alessandra Bisi[1], Silvia Gobbi[1], Angela Rampa[1], Barbara Canonico[3], Mariele Montanari[3], Dmitri V. Krysko[4], Stefano Papa[3], Carmela Fimognari[2*] and Federica Belluti[1*]

[1] Department of Pharmacy and Biotechnology, Alma Mater Studiorum-University of Bologna, Via Belmeloro 6, 40126 Bologna, Italy; [§]Present address: Department of Drug Discovery and Development, Istituto Italiano di Tecnologia, via Morego n.30, 16163 Genova, Italy.

[2] Department for Life Quality Studies, Alma Mater Studiorum-University of Bologna, Corso d'Augusto 237, 47921 Rimini, Italy.

[3] Department of Biomolecular Sciences, University of Urbino Carlo Bo, Urbino, Italy.

[4] Cell Death Investigation and Therapy Laboratory, Department of Human Structure and Repair, Ghent University, Ghent, Belgium; Cancer Research Institute Ghent, Ghent, Belgium; Department of Pathophysiology, Sechenov First Moscow State Medical University, Moscow, Russia.

^These authors contributed equally to this work.

* Correspondence: federica.belluti@unibo.it; Tel.:+39 0512099732 (FB) + carmela.fimognari@unibo.it; Tel.:+390541-434-658 (CF)

1. Structural Characterization of Compound 1.

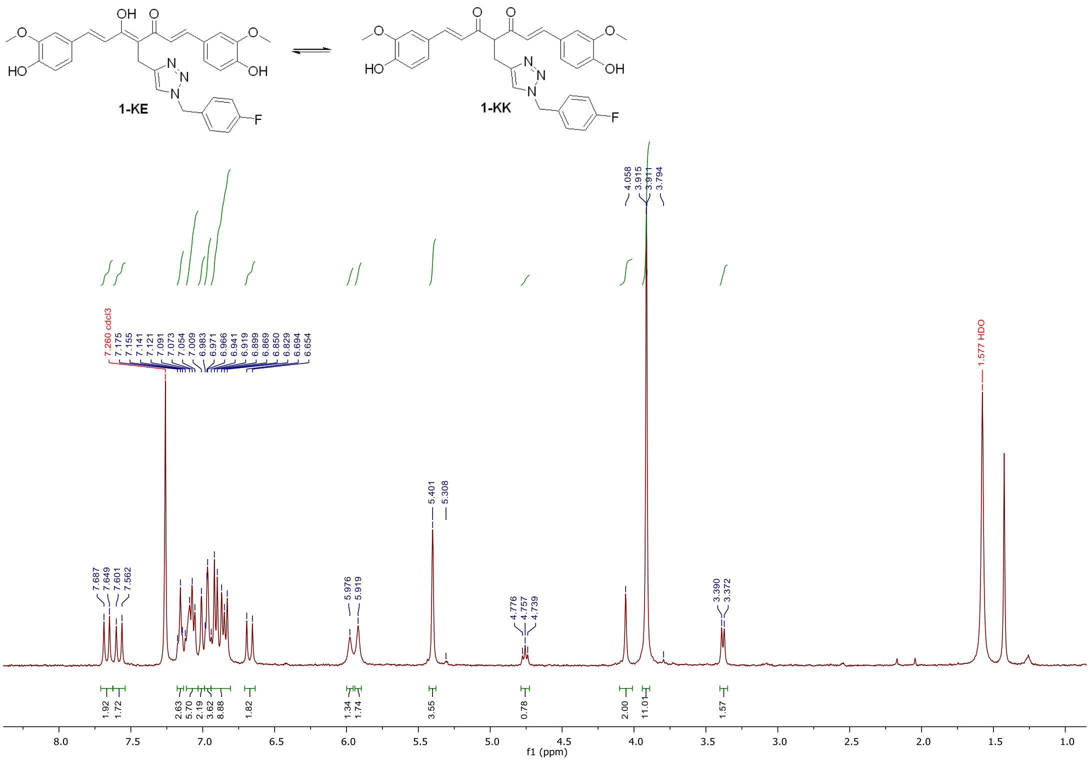

Figure S1. ^{1}H NMR ($CDCl_3$, 400 MHz) copy of compound **1**.

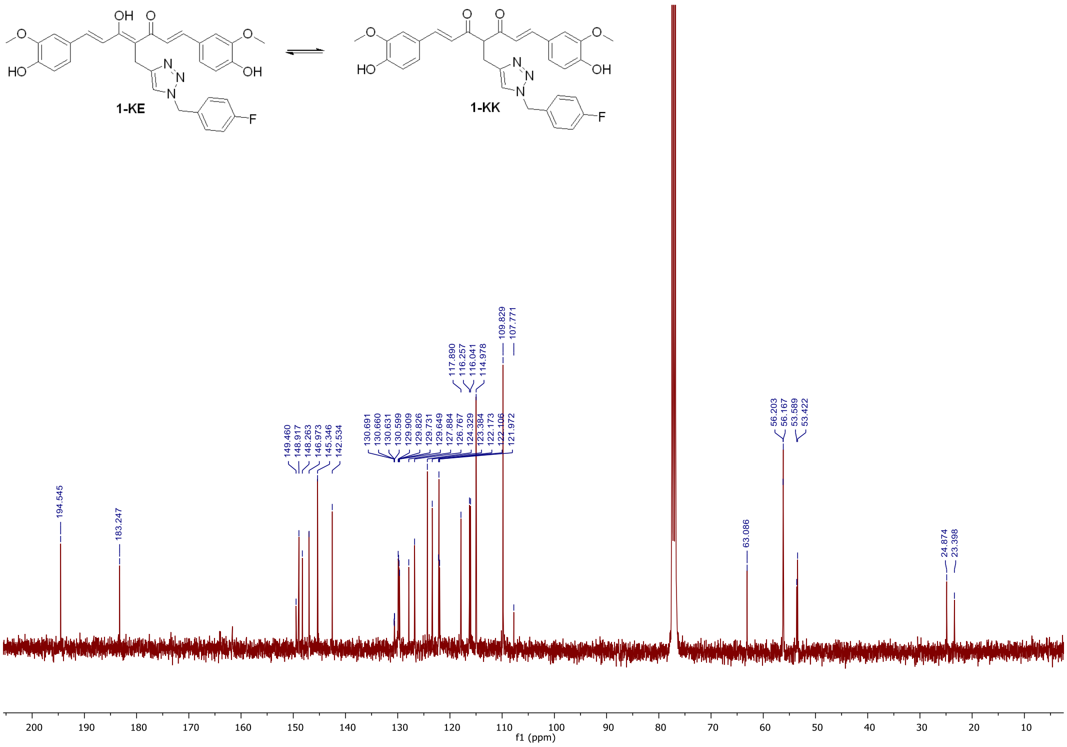

Figure S2. ^{13}C NMR ($CDCl_3$, 101 MHz) copy of compound **1**.

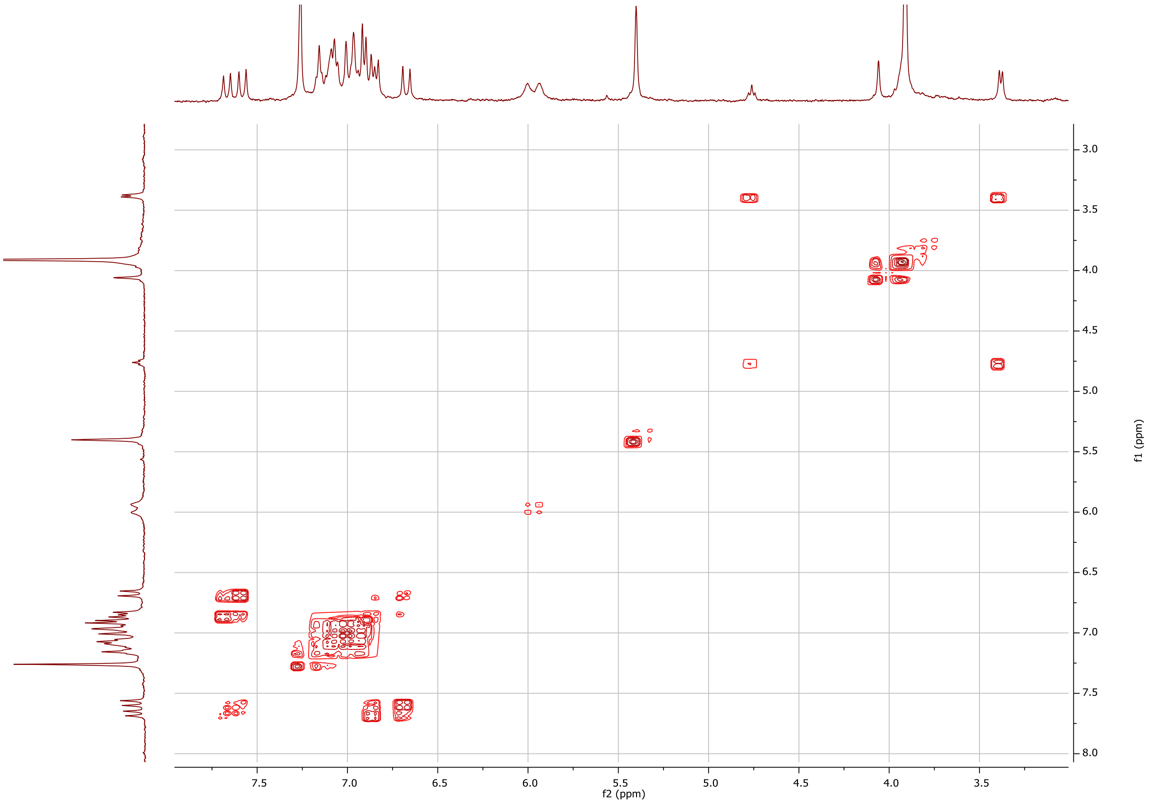

Figure S3. ^{1}H–^{1}H COSY copy of compound **1**.

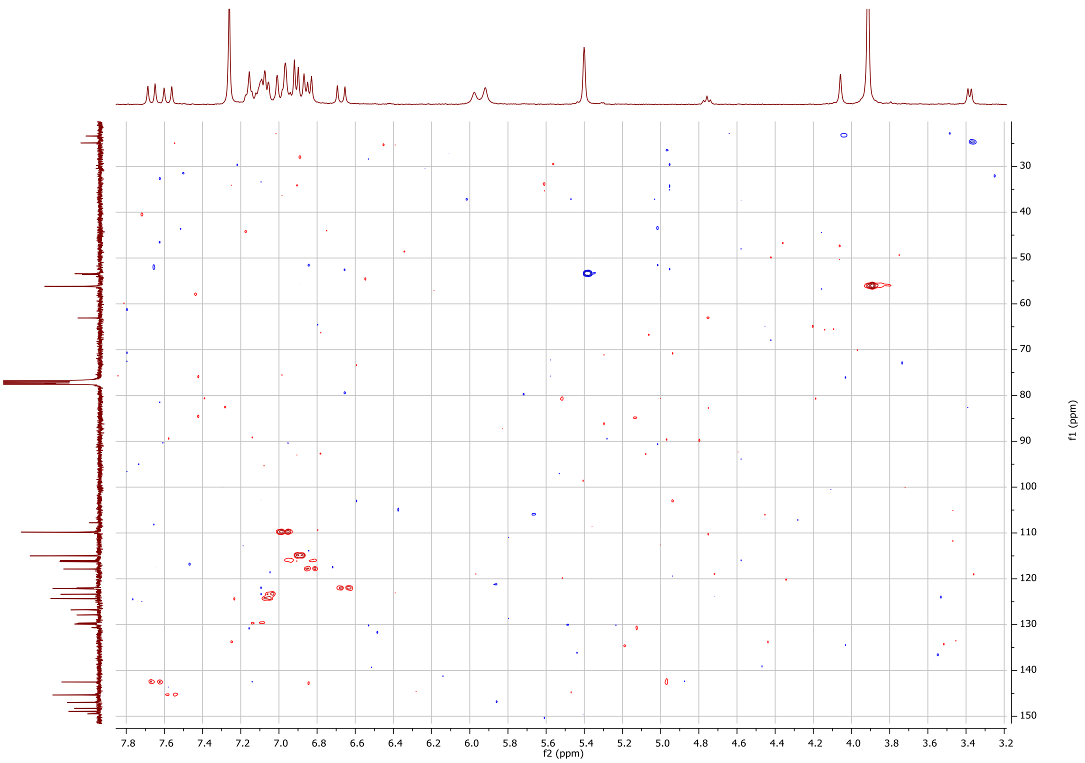

Figure S4. 1H–13C HSQC copy of compound **1**.

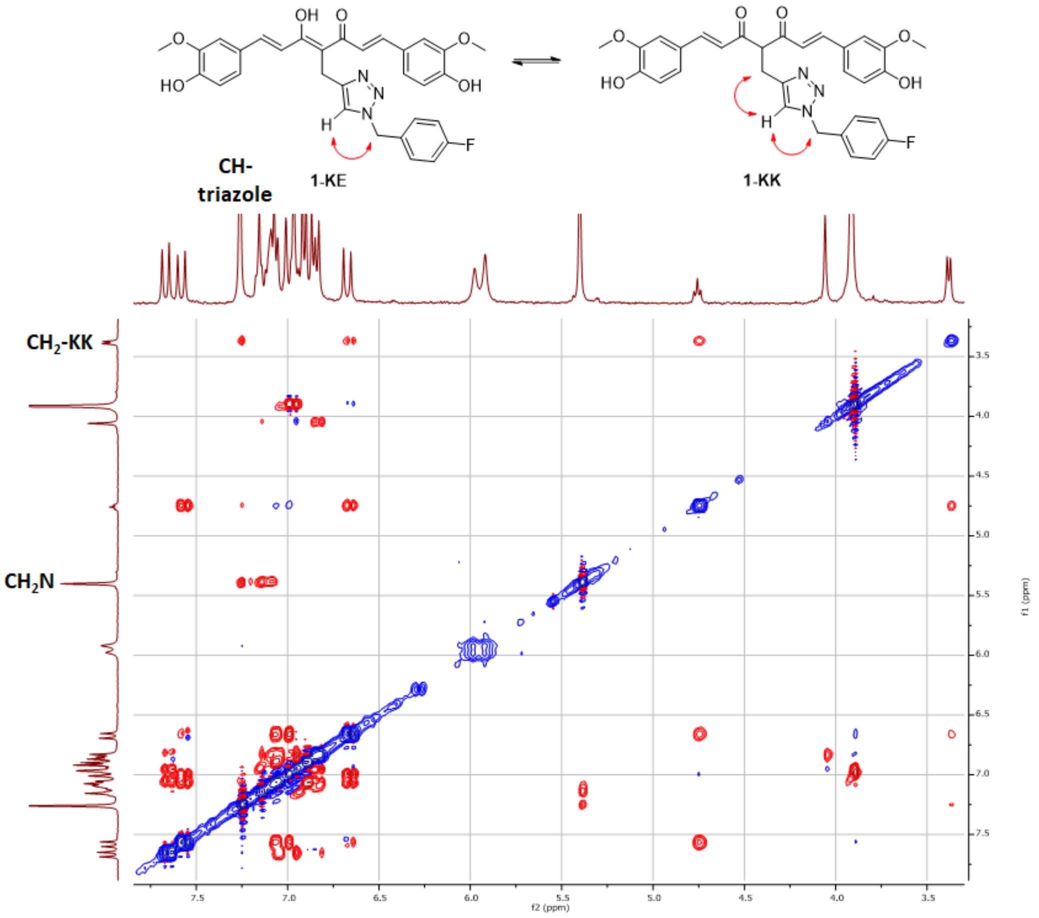

Figure S5. 2D NOESY copy of compound **1**.

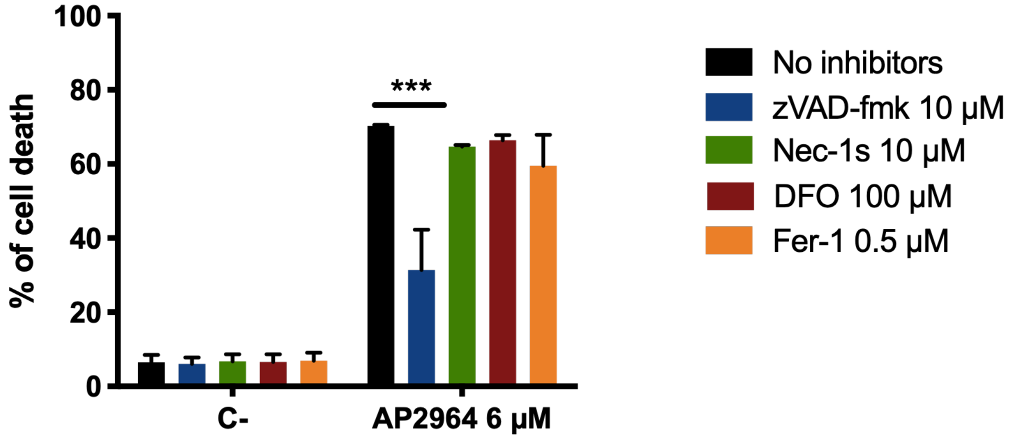

Figure S6. Cell-death analysis of Jurkat cells (% of dead cells) pre-treated for 1h with zVAD-fmk, Nec-1s, DFO, or Fer-1 and then treated with compound **1** for 48h. *** P < 0.001.

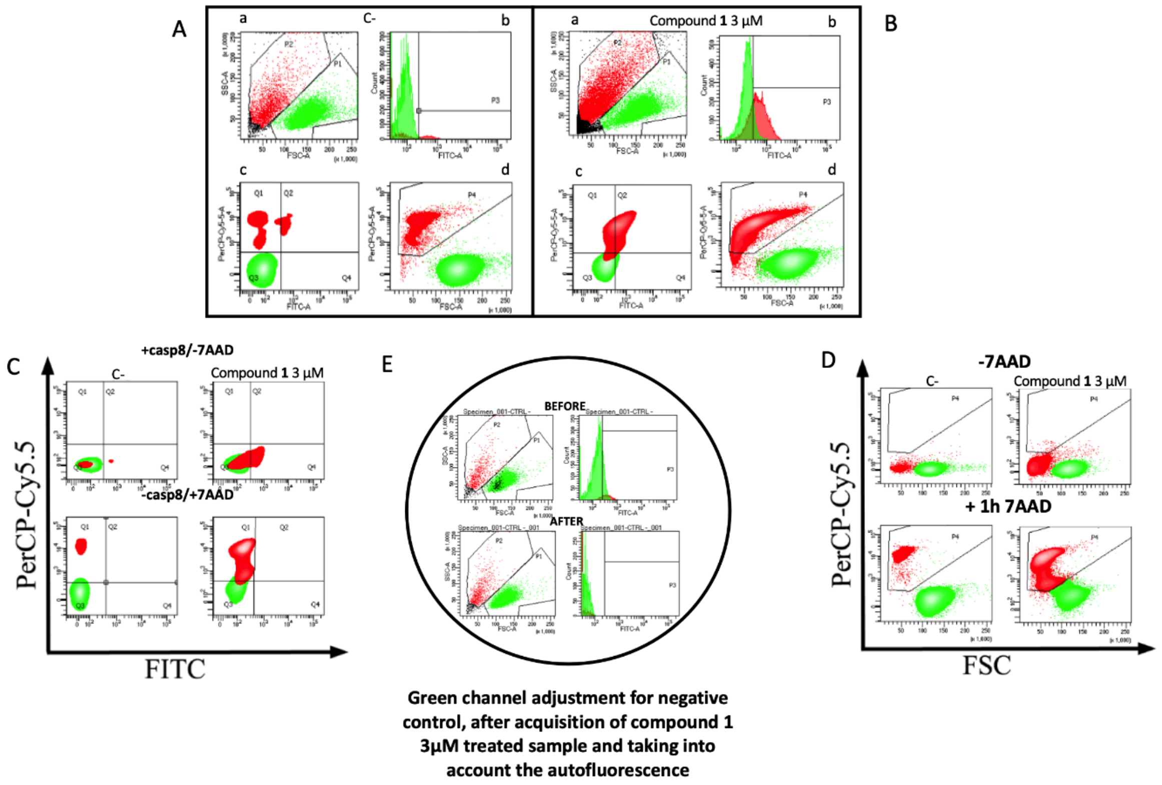

Figure S7. Gating strategy (**A**, **B**, **C**, **D**) and green autofluorescence management (**E**) in caspase 8 analysis. (**A**) and (**B**) show the sequence of dot plots, histograms and contour plot of control and compound **1**-treated samples, respectively. CCRF-CEM were split into different sub-populations depending on the morphologic parameters: red gate shows dead cells, green gate shows viable cells. Black represents debris which were excluded for the analysis. Dot plots in the picture show control (**Aa**) untreated cells and cells treated with the compound **1** for 24 h (**Ba**). In particular, (**Ba**), highlights the huge increase of red events, *i.e.* shrunken virtually apoptotic cells. To clear identify the apoptotic process, 7-AAD and Caspase 8 labelling were performed: histograms (**Ab**) and (**Bb**) show caspase 8 positivity (very low in control cells, while relevant in compound **1**-3 μM-treated cells); contemporary contour plots (**Ac)** and (**Bc)** highlights caspase 8+/7-AAD+ cells. Finally, the

contour plot FSC vs PerCpCy5.5 demonstrates how 60 minutes of incubation at RT for 7-AAD well discriminate between apoptotic (7-AAD^{low}) and necrotic-or late apoptotic (7-AAD^{bright}) cells. Since compound **1** emits fluorescence, the test required several steps and checkpoints, however it was possible to perform it. In (**C**), contour plots FITC vs PerCpCy5.5 are shown for control and compound **1**-treated samples: the first-row highlights both samples only for caspase 8 labelling, the second row only for 7-AAD uptake. Furthermore, (**D**) underlines, for control and compound **1**-treated samples, the positioning of the area of 7-AAD positivity (for both 7AAD^{low} and 7AAD^{bright} events), taking into account distribution of events in unlabeled samples (the upper row). Finally, in (**E**), as stated in the figure, the step of green channel adjustment.

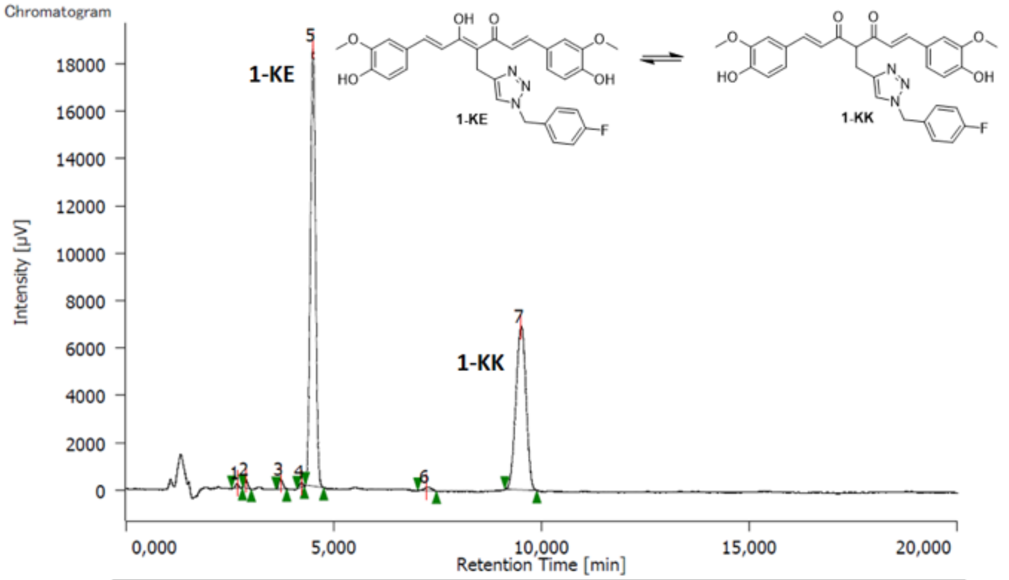

#	Peak Name	CH	tR [min]	Area [µV·sec]	Height [µV]	Area%	Height%	Quantity	NTP	Resolution	Symmetry Factor	Warning
1	Unknown	1	2,687	1010	203	0,352	0,759	N/A	6064	1,436	0,889	
2	Unknown	1	2,897	1939	349	0,677	1,303	N/A	5570	4,757	1,090	
3	Unknown	1	3,730	2928	420	1,021	1,568	N/A	5793	2,716	1,031	
4	Unknown	1	4,233	1185	189	0,414	0,704	N/A	9297	1,353	0,794	
5	Unknown	1	4,507	159922	18584	55,787	69,352	N/A	6171	10,200	1,011	
6	Unknown	1	7,240	1912	158	0,667	0,591	N/A	8847	5,939	1,110	
7	Unknown	1	9,513	117766	6893	41,082	25,724	N/A	6851	N/A	0,961	

Figure S8. HPLC copy for compound **1**.

Physicochemical Properties Prediction. The online server FAFDrugs4 (server available at http://fafdrugs4.mti.univ-paris-diderot.fr) was used to predict physicochemical properties of compounds **1-6**, including logP, logD (at pH 7), flexibility, aqueous solubility (logSw), number of rotatable bonds, hydrogen-bond acceptors (HBAs), and hydrogen-bond donors (HBDs). FAFDrugs4 was also applied to predict the filter PAINS elements among compounds **1-6**. The screening was performed using all three available PAINS filters (PAINS filter A, B and C). None of the screened target compounds contained a PAINS substructure.

Table S1. Physicochemical properties prediction for compounds **1-6**

	logP	logD	logSw	RotatableB	RigidB	Flexibility	HBD	HBA	HBD_HBA
**1**	5.41	5.37	-6.23	11	27	0.29	3	9	12
**2**	5.28	5.08	-6.14	12	27	0.31	3	10	13
**3**	5.33	5.68	-6.0	11	27	0.29	2	8	10
**4**	5.2	5.38	-5.92	12	27	0.31	2	9	11
**5**	6.67	7.74	-7.61	15	38	0.28	1	10	11

**6**	6.41	7.14	-7.44	17	38	0.31	1	12	13

Table 2. Physicochemical properties prediction for compounds **1-6**

**ù**	**Rings**	**MaxSize Ring**	**NumCharges**	**TotalCharge**	**HeavyAtoms**	**CarbonAtoms**	**HeteroAtoms**	**ratio H/C**	**LipinskiViolation**
**1**	4	6	0	0	41	31	10	0.32	2
**2**	4	6	0	0	42	32	10	0.31	2
**3**	4	6	0	0	39	30	9	0.30	2
**4**	4	6	0	0	40	31	9	0.29	2
**5**	6	6	0	0	51	39	12	0.31	2
**6**	6	6	0	0	53	41	12	0.29	3

Table S3. PAINS filters for compounds **1-6**

	**Solubility ForecastIndex**	**Oral Bioavailability VEBER**	**Oral Bioavailability EGAN**	**Phospholipidosis**	**Fsp3**	**PAINS Filter A**	**PAINS Filter B**	**PAINS Filter C**	**Result**
**1**	Reduced Solubility	Low	Low	NonInducer	0.13	0	0	0	Accepted
**2**	Reduced Solubility	Low	Low	NonInducer	0.16	0	0	0	Accepted
**3**	Reduced Solubility	Good	Good	NonInducer	0.10	0	0	0	Accepted
**4**	Reduced Solubility	Good	Good	NonInducer	0.13	0	0	0	Accepted
**5**	Reduced Solubility	Good	Good	NonInducer	0.10	0	0	0	Accepted
**6**	Reduced Solubility	Low	Low	NonInducer	0.15	0	0	0	Accepted
